# The Human Melanoma Proteome Atlas—Complementing the melanoma transcriptome

**DOI:** 10.1002/ctm2.451

**Published:** 2021-07-22

**Authors:** Lazaro Hiram Betancourt, Jeovanis Gil, Aniel Sanchez, Viktória Doma, Magdalena Kuras, Jimmy Rodriguez Murillo, Erika Velasquez, Uğur Çakır, Yonghyo Kim, Yutaka Sugihara, Indira Pla Parada, Beáta Szeitz, Roger Appelqvist, Elisabet Wieslander, Charlotte Welinder, Natália Pinto de Almeida, Nicole Woldmar, Matilda Marko‐Varga, Jonatan Eriksson, Krzysztof Pawłowski, Bo Baldetorp, Christian Ingvar, Håkan Olsson, Lotta Lundgren, Henrik Lindberg, Henriett Oskolas, Boram Lee, Ethan Berge, Marie Sjögren, Carina Eriksson, Dasol Kim, Ho Jeong Kwon, Beatrice Knudsen, Melinda Rezeli, Johan Malm, Runyu Hong, Peter Horvath, A. Marcell Szász, József Tímár, Sarolta Kárpáti, Peter Horvatovich, Tasso Miliotis, Toshihide Nishimura, Harubumi Kato, Erik Steinfelder, Madalina Oppermann, Ken Miller, Francesco Florindi, Quimin Zhou, Gilberto B. Domont, Luciana Pizzatti, Fábio C. S. Nogueira, Leticia Szadai, István Balázs Németh, Henrik Ekedahl, David Fenyö, György Marko‐Varga

**Affiliations:** ^1^ Division of Oncology Department of Clinical Sciences Lund Lund University Lund Sweden; ^2^ Section for Clinical Chemistry Department of Translational Medicine Lund University Skåne University Hospital Malmö Malmö Sweden; ^3^ 2nd Department of Pathology Semmelweis University Budapest Hungary; ^4^ Department of Dermatology Venerology and Dermatooncology Semmelweis University Budapest Hungary; ^5^ Department of Biochemistry and Biophysics Karolinska Institute Stockholm Sweden; ^6^ Department of Internal Medicine and Oncology Semmelweis University Budapest Hungary; ^7^ Chemistry Institute Federal University of Rio de Janeiro Rio de Janeiro Brazil; ^8^ Clinical Protein Science & Imaging Biomedical Centre Department of Biomedical Engineering Lund University Lund Sweden; ^9^ Department of Molecular Biology University of Texas Southwestern medical center Texas; ^10^ Department of Biochemistry and Microbiology Warsaw University of Life Sciences Warszawa Poland; ^11^ SUS University hospital Lund Lund Sweden; ^12^ Department of Surgery Clinical Sciences Lund University Lund Sweden; ^13^ Chemical Genomics Global Research Lab Department of Biotechnology College of Life Science and Biotechnology Yonsei University Seoul Republic of Korea; ^14^ Department of Pathology University of Utah Salt Lake City Utah; ^15^ Department of Biochemistry and Molecular Pharmacology Institute for Systems Genetics New York University Grossman School of Medicine New York City New York; ^16^ Synthetic and Systems Biology Unit Biological Research Center Szeged Hungary; ^17^ Department of Bioinformatics Semmelweis University Budapest Hungary; ^18^ Department of Internal Medicine and Oncology Semmelweis University Budapest Hungary; ^19^ Faculty of Science and Engineering Department of Analytical Biochemistry University of Groningen Groningen The Netherlands; ^20^ Translational Science and Experimental Medicine Cardiovascular, Renal and Metabolism IMED Biotech Unit, AstraZeneca Gothenburg Sweden; ^21^ Department of Oncology St. Marianna University School of Medicine Kanagawa Japan; ^22^ 1st Department of Surgery Tokyo Medical University Tokyo Japan; ^23^ HQ ThermoFisher Scientific San Jose California; ^24^ BBMRI‐ERIC HQ Graz Austria; ^25^ Department of Plastic and Reconstructive Surgery Shanghai Ninth People's Hospital Shanghai Jiao Tong University School of Medicine Shanghai China; ^26^ Department of Dermatology and Allergology University of Szeged Szeged Hungary

**Keywords:** acetylation stoichiometry, BRAF, driver mutations, histopathology, metastatic melanoma, phosphorylation, posttranslational‐modification, proteogenomics

## Abstract

The MM500 meta‐study aims to establish a knowledge basis of the tumor proteome to serve as a complement to genome and transcriptome studies. Somatic mutations and their effect on the transcriptome have been extensively characterized in melanoma. However, the effects of these genetic changes on the proteomic landscape and the impact on cellular processes in melanoma remain poorly understood. In this study, the quantitative mass‐spectrometry‐based proteomic analysis is interfaced with pathological tumor characterization, and associated with clinical data. The melanoma proteome landscape, obtained by the analysis of 505 well‐annotated melanoma tumor samples, is defined based on almost 16 000 proteins, including mutated proteoforms of driver genes. More than 50 million MS/MS spectra were analyzed, resulting in approximately 13,6 million peptide spectrum matches (PSMs). Altogether 13 176 protein‐coding genes, represented by 366 172 peptides, in addition to 52 000 phosphorylation sites, and 4 400 acetylation sites were successfully annotated. This data covers 65% and 74% of the predicted and identified human proteome, respectively. A high degree of correlation (Pearson, up to 0.54) with the melanoma transcriptome of the TCGA repository, with an overlap of 12 751 gene products, was found.

Mapping of the expressed proteins with quantitation, spatiotemporal localization, mutations, splice isoforms, and PTM variants was proven not to be predicted by genome sequencing alone. The melanoma tumor molecular map was complemented by analysis of blood protein expression, including data on proteins regulated after immunotherapy. By adding these key proteomic pillars, the MM500 study expands the knowledge on melanoma disease.

## Graphical abstract

The MM500 meta‐study aims to establish a knowledge basis of the tumor proteome to serve as a complement to genome and transcriptome studies. The melanoma proteome landscape, obtained by the analysis of 505 well‐annotated melanoma tumor samples, is defined based on almost 16 000 proteins, including mutated proteoforms of driver genes. This data covers 65% and 74% of the predicted and identified human proteome, respectively.

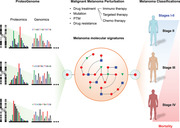

## 1 INTRODUCTION

Malignant melanoma is the deadliest of skin cancers^1^. Incidence has increased dramatically over the past three decades, outpacing almost all other cancers.^2^, ^3^, ^4^ Early diagnosis and surgical excision cures most patients; however, some patients suffer from metastatic disease with a poor prognosis. During the last decade, modern drugs have dramatically improved the outcome with a median survival increasing from months to years.^5^, ^6^, ^7^, ^8^, ^9^

The development of kinase inhibitors targeting the mutated serine/threonine‐protein kinase BRAF, such as vemurafenib, dabrafenib, and encorafenib, have provided significant improvement. Mutations located at BRAF position 600, where the V600E accounts for 90% of the cases, have been associated with increased tumor proliferation, mainly by dysregulation of MEK/ERK receptors.^10^, ^11^, ^12^ The BRAF inhibitors have been combined with cobimetinib, trametinib, and binimetinib that target MEK, another member of the mitogen‐activated protein kinase (MAPK) signaling pathway. This treatment modality has led to improved overall and progression‐free survival.^13^, ^14^, ^15^, ^16^

Parallel advances of the understanding of molecular mechanisms of T cell activation and inhibition and immune homeostasis allowed for the development of checkpoint inhibitors.^17^, ^18^ The therapy targets key regulators of the immune system that restrain T cells from full and persistent activation and proliferation under normal physiologic conditions, but are used by cancer cells to evade the immune response. The best‐known examples are monoclonal antibodies that block CTLA‐4 and PD‐1. These were the first class of therapies shown to improve the overall survival for patients with advanced melanoma, with long‐term, durable tumor regression becoming a reality for some patients.^19^

The existing drug treatments outlined above can prolong survival in metastatic melanoma in more than 50% of patients.^20^, ^21^ However, the majority of patients relapse, due to lack of response and development of resistance. The resistance may develop due to multiple mechanisms, such as tumor cells evading inhibition by promoting alternative survival pathways, mutational events, and changes in the tumor microenvironment.^22^, ^23^, ^24^, ^25^ Clonal expansion due to inherent tumor heterogeneity is important in the context of resistance development.^26^, ^27^, ^28^, ^29^, ^30^

**HIGHLIGHTS**

- A melanoma proteome landscape, complementing genome and transcriptome studies.
- Mass‐spectrometry‐based analysis of almost 16 000 tumor proteins, PTM variants, driver mutations, and missing proteins, reaches 65% and 74% of the predicted and identified human proteome, respectively.
- Identification of proteins regulated after therapy and introduction of the first plasma proteome profile of melanoma patients
- The study contributes to expand melanoma disease understanding.

The Cancer Genome Atlas (TCGA) recently presented a genomic and transcriptomic study with an implication and impact of mutation and genomic classification of cutaneous melanoma^31^ (https://www.cancer.gov/about‐nci/organization/ccg/research/structural‐genomics/tcga).^32^ However, mapping the expressed proteins with quantitation, spatiotemporal localization, mutations, splice isoforms, and posttranslational modifications (PTMs) cannot be predicted by genome sequencing alone.^1^ In the present MM500 study, we outline together with the TCGA transcript expressions, the proteogenomic signature map generated from 505 well‐annotated melanoma samples. This achievement will also allow the development of open‐source bioinformatics tools to access and further mining the data by the scientific community.

## 2 RESULT AND DISCUSSION

This publication belongs to a series of two on the Human Melanoma Proteome sent for publication in Clinical and Translational Medicine. Both are integral parts of the MM500 study. The other manuscript is entitled “The Human Melanoma Proteome Atlas—Defining the Molecular Pathology”. It describes the anatomical sites from which the tumors were isolated, the clinicohistopathological features of the cohort, a detailed histological characterization of the samples, and introduces the protein profiles of analyzed melanoma tumors including the chromosomal and cellular localization, as well as the differential expression of proteins in melanoma cultured cell lines and in tissues with high levels of tumor cells or stroma.

The present proteogenomic melanoma study integrates a comprehensive proteomic analysis with the genomic data from TCGA. The mass spectrometry‐based proteomics is based on the amino acid sequence of all proteins expressed in patient tumors. The results from the MM500 study are dependent on the detailed knowledge of the human genome and its modifications in melanoma with a direct bearing on protein function.^33^, ^34^

**FIGURE 1:**
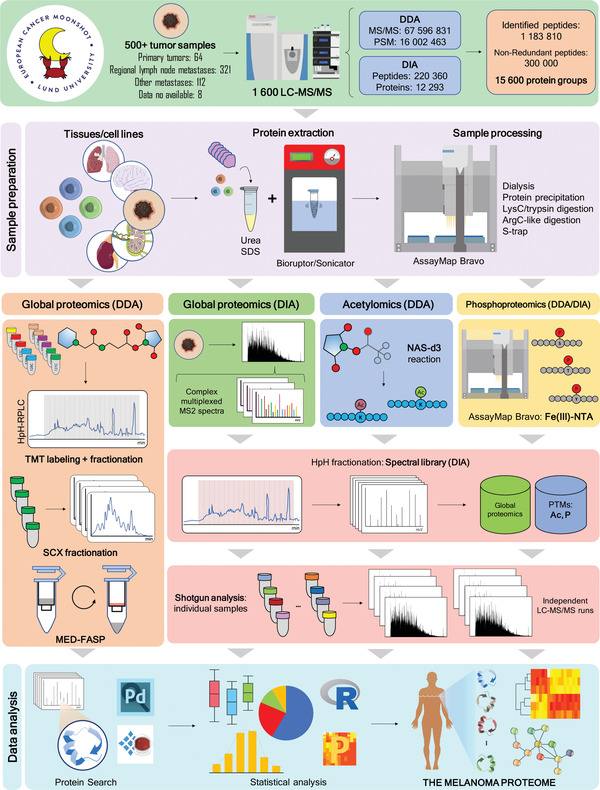
Comprehensive view of proteomic workflows used in the MM500 study. (Upper panel) 505 melanoma tissue samples and four cultured cell lines were analyzed. 1549 LC‐MS/MS experiments produced a proteomic signature of melanoma based on the quantification of 15 973 protein groups representing more than 360 000 nonredundant peptides. (Sample preparation) Several protocols were used which included protein extraction in the presence of urea or SDS with the aid of a Sonifier or a Bioruptor, followed by manual or automatic enzymatic digestion. (Global proteomics) This was performed using both DDA and DIA. DDA data was generated by TMT 11‐plex technology combined with high pH RP‐HPLC fractionation; by SCX stepwise separation of peptide mixtures, by the analysis of fractions derived from the MED‐FASP method, and also by shotgun proteomics. (Acetylomics) DIA‐MS was used to determine naturally occurring protein acetylation sites. This was achieved by modifying protein‐free lysine e‐amino groups with deuterium‐labeled acetyl groups, which upon MS peptide identification and quantitation allowed distinguishing chemically labeled acetylation from endogenous acetylation.^80^ (Phosphoproteomics) Enrichment of phosphopeptides was performed in the Bravo AssayMap robot^110^ and isolated phosphopeptides were directly analyzed by DDA or DIA. (Spectral Libraries of DIA‐MS) MS/MS spectral libraries for DIA‐MS global proteomics acetylomics and phosphoproteomics were built out of DDA‐LC‐MS/MS data. This included shotgun analysis of the very same samples submitted to DIA‐MS, of other samples from melanoma tissues and cultured cells used in this meta‐study, as well as the analysis of a mixture of these samples previously fractionated by high pH RP‐HPLC. (Shotgun analysis) Individual samples were submitted to LC‐MS/MS analysis either in DDA or DIA modes. (Data analysis) The programs Proteome Discoverer and Spectronaut were used throughout all the experiments for protein identification and quantitation

The workflow process undertaken in the MM500 study includes tumor tissue handling, sample preparation, LC‐MS/MS analysis, and data processing are outlined in Figure 1. This molecular pathology process workflow has been extensively automated with high‐end technology platforms.

### 2.1 Global quantitation of the melanoma proteome

**FIGURE 2:**
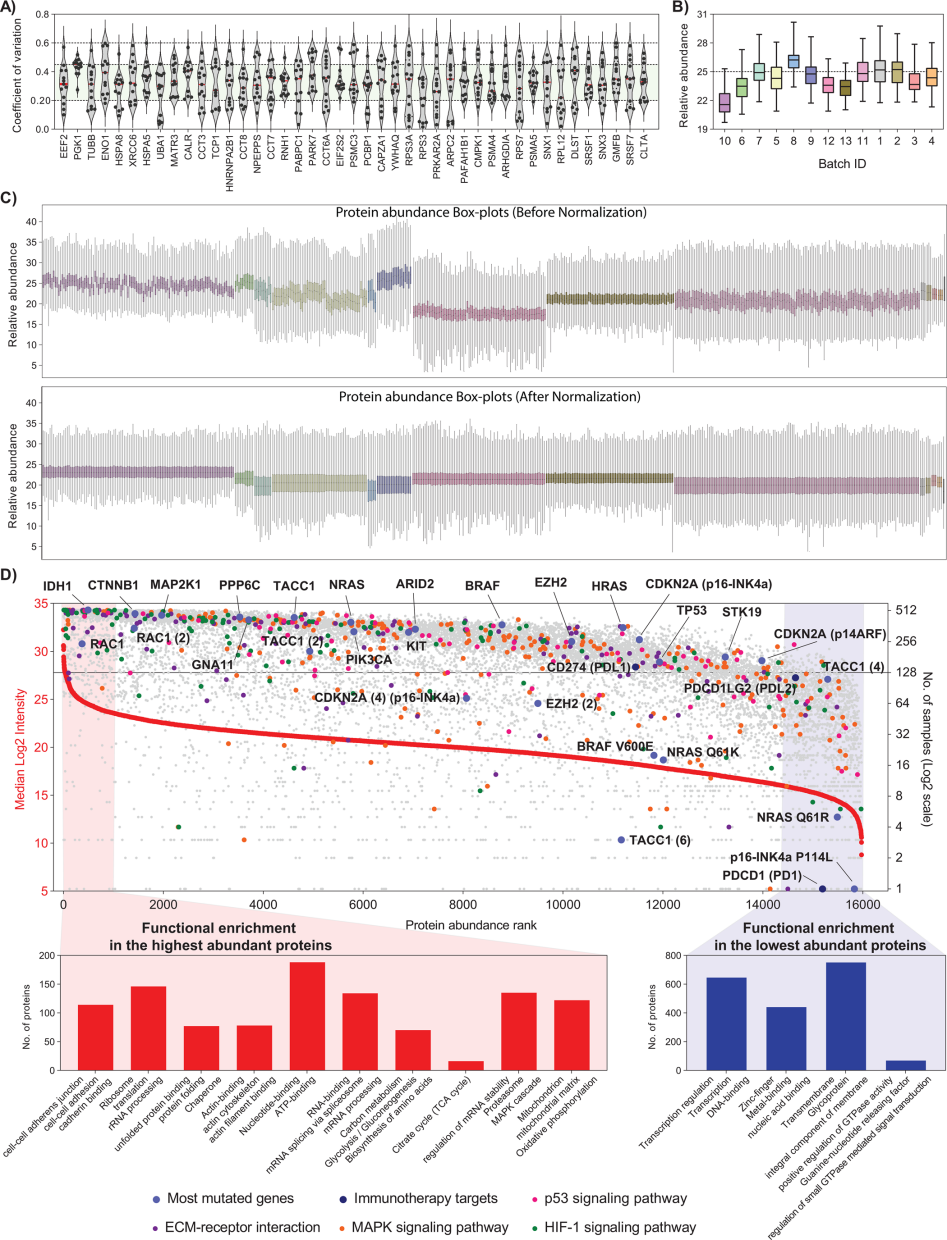
The Melanoma Protein Abundance Map. LC‐MS/MS data was first normalized across batches of analysis in the MM500 study. (A) Violin plots showing the distribution of intrabatch coefficients of variation for the 45 proteins, identified in 100% of the samples and with less than 60% of variation in all batches. (B) Box plots of the relative abundance of the 45 less variable proteins in each batch. The median abundance in each batch was used for inter‐batch abundance correction of the melanoma proteome. (C) Box plots of protein relative abundance across all samples of the the study, before (top panel) and after (bottom panel) intra‐ and interbatch abundance normalization using the 45 proteins with the lowest variability. (D) Distribution of the malignant melanoma proteome ranked according to protein abundance across all samples (left *y*‐axis) and the number of samples where the protein was identified (right *y*‐axis). Proteins were represented by the gene names. The lines point to WT protein products of genes with driver mutations in melanoma. Proteins involved in pathways commonly dysregulated in melanoma, proteins with known driver mutations, and proteins linked to melanoma therapy are marked in different colors as indicated. The number in parentheses specifies the designated isoform of the protein. A typical example is the protein Transforming acidic coiled‐coil‐containing protein 1, where the canonical protein TACC1, the isoform 2 TACC1(2), and isoform 4 TACC1(4) were quantified. A more complex example is represented by the gene CDKN2A that codes for the canonical proteins p16‐INK4a and p14ARF being both quantified, together with the isoform 4 of the former (CDKN2A (4) p16‐INK4a) and the mutated protein p16‐INK4a P114L. At the edges of the plot are highlighted enriched pathways for high‐ (red) and low‐ (blue) abundance proteins

The MM500 cohort was processed and analyzed in subsequent sample batches for both, global proteomic and phosphoproteomic quantitative studies. These multiple data sets were combined to estimate a median abundance for every protein. Raw abundance measurements were first log2 transformed and the median value for all the proteins in each sample was subtracted. Next, 45 proteins with the lowest variability (CV < 60%) and commonly identified across all samples were selected (Figures 2A and 2B). These proteins were strongly correlated with biological processes and molecular functions that primarily included regulation of cellular component organization, regulation of protein localization, and cytoskeleton organization; indeed, acting as housekeeping proteins. Protein abundance normalization was then performed in each batch of analysis by subtracting the median abundance for these 45 proteins. The box‐plots of the protein abundances of all the samples before and after the normalization procedure are illustrated in Figure 2C. The data showed a good level of normalization that adequately corrected for different sample processing or other technical biases. Finally, the protein relative abundances in the melanoma proteome were estimated taking into account the median abundance across the MM500 in the normalized dataset.

The procedure described above enabled ranking of all identified proteoforms in the global proteomics and the PTMs analysis based on their relative abundance in melanoma. In total 15 973 identified proteoforms were plotted, including the mutated proteins BRAF, NRAS, and CDKN2A (Figure 2D and Table S1). This analysis enabled direct positioning of the protein expression of melanoma driver mutations^35^ with wild‐type (WT) proteins and verification on the frequency of detection within the tumors isolated from patients. WT IDH1 and WT RAC1 had the highest expression and were present in almost all tumor samples. The WT variants of BRAF, TP53 and the subunits p16‐INK4a and p14ARF of CDKN2A were quantified in 362 (72%), 152 (30%), 256 (51%), and 159 (32%) of the tumor samples, respectively. On the other hand, proteins bearing driver mutations, including BRAF V600E, NRAS Q61K/R, and p16‐INK4a P114L, had lower abundance and were identified in considerably fewer samples than the corresponding WT proteins (Figure 2D). It also became apparent that in discovery proteomics the detection of key mutations in melanoma can only be achieved through deep mining experiments where 10 000 or more proteins are identified. Overall, the data output presented, displays the expressed protein abundance of melanoma in a range of approximately six orders of magnitude and allow to extensively map and quantify biological pathways dysregulated during melanoma development and progression. The majority of the proteins identified in this study were quantified in a high number of samples (Figure 2D).

The most abundant proteins are involved in key functions in the cell, such as proteins involved in cytosolic ribosome and translation, the cytoskeleton, metabolic pathways such as glycolysis and biosynthesis of amino acids and proteins from the transcription machinery. Besides, the high‐abundance melanoma proteome is significantly enriched in mitochondrial proteins, particularly those linked to the energy production through the TCA cycle and oxidative phosphorylation, highlighting the mitochondrial function dependence. These findings provide evidence to further explore mitochondrial pathways as potential therapeutic vulnerabilities in melanoma.^36^, ^37^, ^38^ Oppositely, the melanoma low abundant proteome is composed by proteins involved in the regulation of transcription and other related processes, and signaling cascades. Not surprisingly a large set of the low‐abundance proteins were reported as integral components of the membrane which are generally difficult to identify due to their hydrophobicity, and they are usually underrepresented in global proteome studies.

### 2.2 RNA‐protein overlap and comparison with the human proteome

**FIGURE 3:**
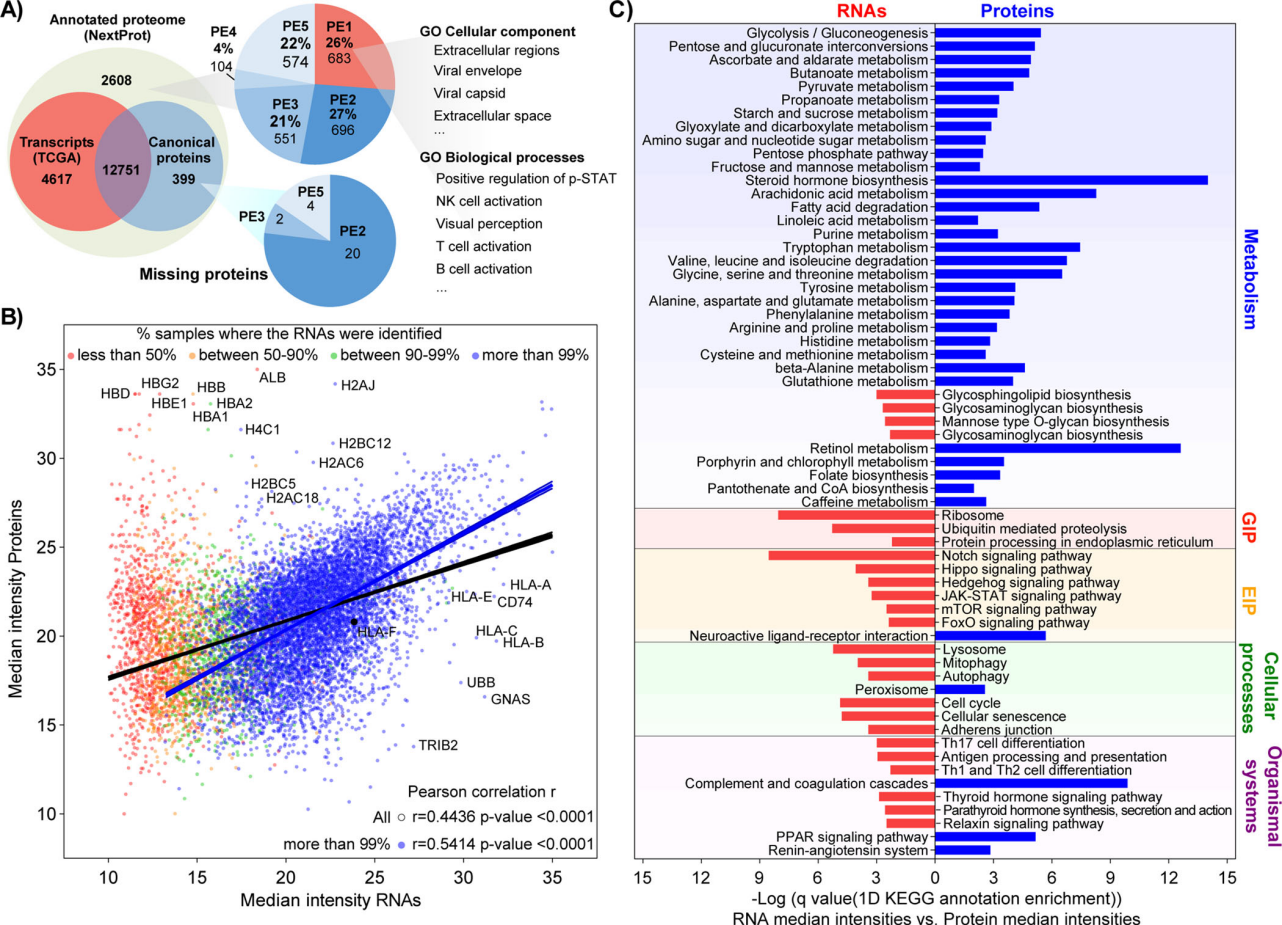
Comparison of MM500 melanoma proteome, TCGA melanoma transcriptome, and the Human proteome. (A) Overlapping of transcripts (TCGÀ), identified melanoma protein‐coding genes (canonical proteins) and the human proteome (NextProt). In NextProt, proteins are categorized in a PE1‐PE5 structure, in acceptance within the scientific community (https://www.uniprot.org/help/protein_existence),^114^ with five types of evidence for the existence of a protein: (1) experimental evidence at protein level; (2) experimental evidence at transcript level; (3) protein inferred from homology; (4) protein predicted; (5) protein is uncertain. (B) Correlation relationships between mRNA and mean protein expressions. Scatter plot of median intensity of the proteins identified in this study versus the median intensity of transcripts coming from RNA sequencing data from 443 melanoma tumors downloaded from the TCGA repositories. RNA sequencing data were classified according to the number of samples where the transcript was detected. The Pearson correlation and best‐fitting curve were provided for the whole dataset and those transcripts quantified in more than 99% of the samples. Both datasets were scaled to the range between 10 and 35. (C) Representation of the 1D KEGG annotation enrichment of the differences between the median intensity in all samples of the transcripts and the proteins. Bars indicate the level of enrichment according to a Benjamini‐Hochberg FDR truncation strategy. Blue correspond to pathways overrepresented for proteins relatively more abundant than their transcripts and Red bars correspond to pathways overrepresented in those transcripts showing relatively more abundance than their corresponding protein. Pathways were sorted based on their KEGG classification. The 1D annotation enrichment analysis was performed under the Perseus platform

The detected 15 973 proteoforms accounted for the identification of 13 176 different protein‐coding genes. These were compared with the available transcriptomic data from 443 melanoma tumor tissues in the TCGA repository. Here, we selected the 17 431 transcripts, corresponding to 17 368 different genes, with at least ten reads from the RNA sequencing (Figure 3A). We found that nearly than 400 protein‐coding genes identified in this study were not detected at the transcript level. These set of “orphan” proteins were plotted based on their abundance and number of samples that where they were detected (Figure S1A). The results showed that most of these proteins were identified in large number of samples and across a large range of abundances. The functional annotation enrichment analysis and protein interaction network reveal that these proteins mostly come from mitochondrial genome coded proteins, the extracellular space and from blood (Figure S1B). Transcripts originated in the mitochondrial matrix were not analyzed in the RNA sequencing data from the TCGA repositories of melanoma tumors (see data and code availability under Materials and methods). The present data on melanoma proteome includes 12 out of the 13 proteins produced in the mitochondria. The identification of proteins acting in the extracellular space could be attributable to the diverse tissue compositions between the TCGA and the MM500 cohorts, since we did not impose any filter in the cell content of the samples. The blood protein origin for some of the proteins was confirmed by the detection of more than 100 of these proteins (mostly antibodies) in a pool of blood plasma of melanoma patients (see Section 2.8). The absence of transcript counterparts in the TCGA dataset for antibodies, histones, and proteins of the MHC complex I/II could also be explained by the sequence variabilities of these proteins across individuals. Interestingly, HLA proteins are also known to be heavily mutated in several cancers and particularly in melanoma.^39^, ^40^ Moreover, the exclusion of transient transcripts with less than 10 reads from the TCGA dataset, should also be considered, which was the filter applied to the RNA sequencing data.

The RNA sequencing dataset contains 4617 transcripts that had no protein counterpart in the MM500 melanoma data, which could indicate that a fraction of the melanoma transcriptome has very low or absent translation, or tight regulation of their protein stability. This observation can also be partially explained by the fact that these datasets were derived from different tumor cohorts, suggesting the expression of a fraction of the melanoma proteome not captured within the MM500 meta‐study. These analyses were contrasted with the 20 350 annotated human genes (Figure 3A). It was found that 2608 proteins included in the full human proteome were not identified in the present melanoma data, nor were any corresponding transcripts detected in the TCGA data. Most of these proteins (74%) are part of the so‐called missing proteins and classified as PE2 (696 proteins with evidence at the transcript level in other studies), PE3 (551 proteins with sequence similarities), PE4 (104 proteins with in silico prediction) and PE5 (551 proteins derived from pseudogenes or with dubious information). The remaining 26% are classified as PE1; that is, they do have strong experimental evidence supporting their identification. These results should be put into perspective to the entire MM500 study. The 13 176 protein‐coding genes identified in melanoma samples covered 65% and 74% of the predicted and identified human proteomes, respectively. Besides, when complemented with the TCGA data, altogether transcriptomic and proteomic data in melanoma have provided evidence for 87.3% and 99.4% of the predicted and the identified human proteomes, respectively.

### 2.3 MM500—NextProt and TCGA database annotations from melanoma tumors

Next, the protein relative abundance from MM500 global proteomics (15 530 proteoforms, 12 878 different genes) was compared with the melanoma tumors mRNA expression levels from the TCGA repository. The relative abundance of the transcripts in melanoma was calculated based on the mean across all the samples, similar to the proteomic data in the MM500 study. A total of 12 751 gene products were commonly identified in both datasets. By plotting the abundance of proteins and transcripts a significant positive correlation of 0.44 was observed. This result is in line with previous findings on protein‐mRNA expression correlation in mammalian cells.^41^ Moreover, when taking into account transcripts detected in 99% of the samples the correlation rises to 0.54 (Figure 3B). Despite the high correlation, some proteins showed a disproportional higher abundance than their corresponding transcripts. Not surprisingly, in this group we found proteins from blood, for example albumin and all subunits of hemoglobin, represented in Figure 3B. Also, most of the histone variants were overrepresented in the melanoma proteome, which is indicative of the low clearance rate of these proteins. Interestingly, several MHC protein elements were underrepresented in relation to their corresponding transcripts, highlighting an important aspect of melanoma development and progression by modulating the antigen presentation at the protein level.

To better understand the disparities between the melanoma transcriptome and proteome a functional annotation enrichment analysis using the differences in abundance between proteins and transcripts was performed. According to the KEGG pathways annotations, the melanoma proteome is overrepresented in most of the metabolic pathways, particularly, those linked to energy and proliferation intermediates production, including the metabolism of amino acids. Oppositely, when compare to the transcriptome, the melanoma proteome is underrepresented in genetic and environmental information processing related pathways, including signaling pathways and cellular processes (Figure 3C). The antigen processing and presentation, which plays a critical role in the immune system response, was underrepresented in the proteome. In this sense, the melanoma strategy to downregulate at the translational level the antigen presentation, allows the progression of the disease by evading the immune surveillance.^42^

### 2.4 Missing proteins

Recently, we reported mass spectrometry evidence and associations with cancer‐related functions for 33 novel proteins from well‐characterized 140 metastatic melanoma samples that were also included within the MM500 cohort.^43^ Here, new mass spectrometry data for 26 new “missing proteins were added after the analysis of the 505 melanoma samples” (Table S2). The new proteins are distributed as PE2 (n = 20), PE3 (n = 2), and PE5 (n = 4), (Figure 3A). Three of them were identified with at least two uniquely mapping peptides with length ≥ 9 amino acids (AA), which is in agreement with the Human Proteome Project (HPP) interpretation guidelines for missing proteins (https://www.hupo.org/HPP‐Data‐Interpretation‐Guidelines).^44^ In contrast, 19 (73%) out of these 26 proteins were also identified as transcripts in melanoma tumor samples. Notably, the Small Proline‐rich protein 4 was identified for the first time, with two peptides ≥ 9 AA. In the present study, a total of eight proteins from the family (SPRR1 to SPRR4) were identified, all of them also identified at the RNA level. To the best of our knowledge, there is little evidence of the identification of this family of proteins in melanoma samples.^45^ The SPRRs proteins are encoded by a multigene family clustered within the epidermal differentiation complex on human chromosome 1, and have been associated with the progression of several types of tumors such as colorectal, breast, and brain tumors.^46^, ^47^

**FIGURE 4:**
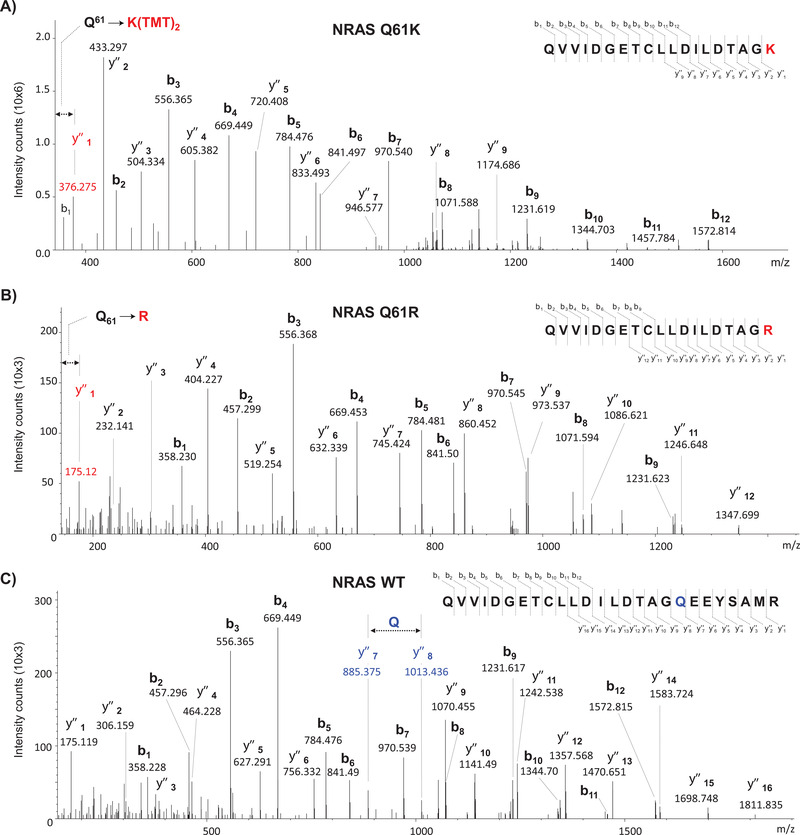
Identification of mutated variants of NRAS and WT NRAS by mass spectrometry. (A) Assigned MS/MS spectrum of the TMT‐labeled peptide QVVIDGETCLLDILDTAGK corresponding to the mutation NRAS Q61K. (B) Assigned MS/MS spectrum of the TMT‐labeled peptide QVVIDGETCLLDILDTAGR corresponding to the mutation NRAS Q61R. (C) Assigned MS/MS spectrum of the TMT‐labeled peptide QVVIDGETCLLDILDTAGQEEYSAMR of WT NRAS. The Q61K/R mutations introduced an additional trypsin cleavage in the sequence of the WT protein, rendering shorter mutated peptides lacking the C‐terminal part (‐EEYSAMR) of the WT peptide sequence

### 2.5 Identification of melanoma protein mutations

Large‐scale genetic studies have provided important landscapes of mutations in melanoma. Mutations may alter the amino acid sequence of the proteins, which in turn can potentially affect the protein folding, stability, abundance, function, interactions with other proteins, subcellular localization and may be related to disease progression. Little is known about the protein expression of mutations in melanoma, most probably due to low abundance and technology limitations.

In melanoma, the main driver mutation, which is responsible for at least 50% of melanomas, is BRAF V600E. BRAF is a kinase that activates the MAPK signaling pathway through the phosphorylation of MAP2K1. Mutated BRAF is constantly activated which promotes proliferation signals in the cell. Other melanoma driver mutations are also involved in the regulation of this pathway, as is the case of mutated NRAS and MAP2K1.

The clinical relevance of the BRAF V600E mutation in melanoma is well known and understood. BRAF V600E mutation analysis at the DNA level in melanoma samples is used to select patients who could respond to BRAF inhibitors.^48^ Noteworthy, the drugs developed are directed toward the mutated protein and not to the corresponding gene. It is not fully known to which extent the BRAF V600E gene is translated into protein and the association between the levels of the target protein and therapy efficacy has not been characterized in detail. Recently our group published data to support a link between BRAFV600E mutated protein and melanoma patient survival.^49^

We explored our ability to identify melanoma key mutations by including amino acid sequences containing known driver mutations of the disease in the database used for protein identification. The applied strategy identified eight of these mutations in six proteins (Table 1, Figures 4 and S2). Except for BRAF V600E (Figure S2E), this result constitutes the first report of identification by mass spectrometry of these mutations at the protein level in melanoma tumor samples.

**TABLE 1:** Summary of mutations identified in this study

Gene	Mutation	Identified peptide^b^	# PSMs^c^
**BRAF**	V600E^a^	IGDFGLATEK	8
**NRAS**	Q61K^a^	QVVIDGETCLLDILDTAGK	12
	Q61R^a^	QVVIDGETCLLDILDTAGR	3
	G12A	LVVVGAAGVGK	1
**KRAS**	G13D	LVVVGAGDVGK	1
**c‐KIT**	N566D	VVEEINGDNYVYIDPTQLPYDHK	1
**CDKN2A**	P114L^a^	LLVDLAEELGHR	1
**GNA11**	N266K	SSVILFLNK	3

^a^ Mutation identification supported by previous genomic studies on the samples.

^b^ Substituted amino acid is highlighted in red.

^c^ Peptide Spectrum Matches indicates the number of MS/MS spectra that were assigned to the mutated peptide.

Four mutations in two members of the RAS family, the small GTPase proteins NRAS and KRAS were identified. Figures 4A, 4B, S2A, and 4C show the MS/MS spectra corresponding to peptides QVVIDGETCLLDILDTAGK^61^, QVVIDGETCLLDILDTAGR^61^, LVVVGA^12^AGVGK and QVVIDGETCLLDILDTAGQ^61^EEYSAMR of NRAS with the mutations Q61K, Q61R, G12A, and the peptide without mutation at Gln61, respectively. NRAS is the second most prevalent oncogene after BRAF in melanoma and has been found mutated in 15%‐30% of cases.^31^ NRAS mutations at positions Gln61 and Gly12 are among the most frequently observed for this gene. They cause an altered GTPase activity that keeps NRAS activated, which induces a constitutive activation of the MAPK pathway with cell proliferation, dysregulation of the cell cycle, and activation of other pro‐survival pathways.^35^ Melanoma patients with mutated NRAS have different features compared to those harboring BRAF mutations: they are older, have a history of UV exposure, have thicker primary tumors, and a higher rate of mitosis.^50^ KRAS mutations have been observed in approximately 2% of cases in cutaneous melanoma. The G13D mutation, detected in the peptide LVVVGGD^13^GVGK (Figure S2B) is rather rare and known to decrease GTP binding and its hydrolysis.^51^ To date, despite the extensive efforts to target these genes, therapeutic inhibition of RAS has failed.

CDKN2A (cyclin‐dependent kinase 2A) is the major high‐penetrance susceptibility gene with germline mutations identified in 20%‐40% of melanoma families.^52^ The CDKN2A gene encodes two proteins, p16 (INK4A) and p14 (ARF), with both function as tumor suppressors by regulating cell growth and survival. We identified the peptide LL^114^VDLAEELGHR correspondng to the mutation P114L in p16‐INK4a (Figure S2C).The p16‐INK4a P114L is one of the most frequently recurring mutations for CDKN2A in melanoma tumors^53^ and it is known to confer a loss of function to the proteins.^54^

We also identified the peptide ^267^SSVILFLNK^268^, which provided indirect evidence of the mutation N266K (Figure S2D) in the highly homologous proteins GNA11 (guanine nucleotide‐binding protein subunit alpha‐11) and GNAQ (Guanine nucleotide‐binding protein G(q) subunit alpha). The unlikely cleavage by trypsin at Ans266 (the preceding amino acid to the identified peptide) suggested the presence of the mutation N266K, which generated a specific cleavage site such as a Lys residue for the enzyme. GNA11 acts as a molecular switch for G‐proteins and plays an important role in the hydrolysis of guanosine triphosphate (GTP).

Mutations in GNA11 have been associated with activation of the MAPK pathway and cell proliferation in uveal melanoma.^55^, ^56^, ^57^, ^58^ Although rare, the occurrence of GNAQ and GNA11 mutations in nonuveal melanoma, like in the present study, has been documented. It has been found that metastatic GNA11 mutant nonuveal melanomas respond poorly to available systemic therapies, including immune checkpoint inhibition, which points to the urgency of novel therapeutic approaches for these tumors.^59^

Finally, we have tentatively assigned the c‐KIT N566D mutation in the peptide VVEEINGD^566^NYVYIDPTQLPYDHK. The c‐KIT gene encodes a tyrosine kinase receptor, involved in both the MAP kinase and AKT pathways, which are intimately involved with cell proliferation and survival.^60^, ^61^ Intracellular signaling through KIT plays a critical role in melanocyte development. For the last ten years, it has had an emerging role as an oncogene and therapeutic target in melanoma.^62^, ^63^, ^64^ KIT mutations are found in only 3% of all melanomas but a disproportionate amount of KIT aberrations has been identified in melanoma arising from chronically sun‐damaged skin in acral and mucosal tissue; the N566D mutation being among the most commonly found in this gene.^65^, ^66^ KIT mutations are nearly always mutually exclusive with NRAS or BRAF and thus define a unique subtype of melanoma. The N566D mutation was detected by automated protein identification but this could not be fully confirmed by manual interpretation of the MS/MS spectrum like in the above‐mentioned mutations.

The detection of the mutations BRAF V600E, NRAS Q61K/R, and CDKN2A‐p16(INK4A) P114L was also supported by previous analysis of DNA and RNA of the tumor samples.^67^, ^68^ This served as validation of the mass spectrometry detection and allowed a precise quantification of these mutations. The results suggest that driver mutations are expressed at a lower level when compared with the constituent proteins of the melanoma proteome map outlined in our study (Figure 2D).

### 2.6 Posttranslational modification (PTM) analysis

Two prevalent covalent posttranslational modifications (PTM) of proteins are phosphorylation on serine, threonine, and tyrosine residues, as well as acetylation of the lysine residues.^69^, ^70^, ^71^ These events are crucial for the cell machinery and signaling pathways, which may include crosstalk between the PTMs and even become key regulators with link to cancer disease.^72^, ^73^, ^74^, ^75^, ^76^, ^77^

#### 2.6.1 Phosphoproteome

**FIGURE 5:**
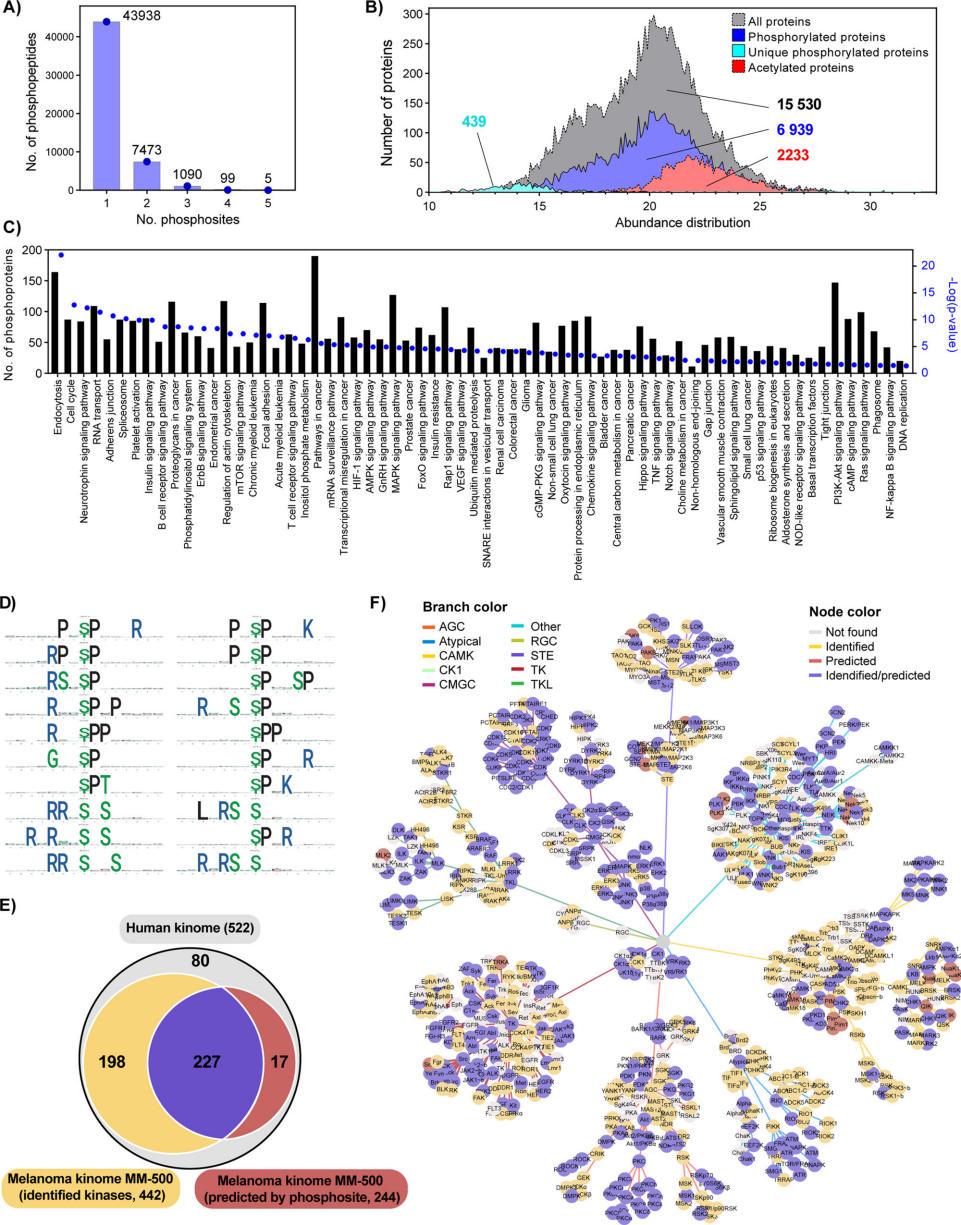
Melanoma phosphoproteome and kinome analysis. (A) Number of identified mono‐, di‐ and multiphosphorylated peptides. (B) Abundance distribution of the melanoma proteome phosphoproteome and acetylome. The relative abundance of the proteins was calculated based on the quantitative proteomic data, with the exception of the 439 proteins that were only detected after phosphopeptide enrichment. In that case the abundance was calculated from the phosphopeptides identified. (C) Distribution of the melanoma phosphoproteome based on enriched KEGG pathway analysis. (D) First 20 phosphorylation motifs in the output list of the motifeR software. (E) Venn diagram of the melanoma kinome comprising the kinases directly identified in this meta‐study and kinases predicted based on detection of phosphorylation motifs of identified phosphosites, both covering a comprehensive part of the human kinome. (F) Kinome network mapping based on direct identification of kinases and computational kinase‐specific phosphorylation site prediction. Kinases identified, predicted, identified/predicted, and not found have different color nodes and are clustered in different categories based on the branch color

The phosphoproteome of 200 melanoma tumor samples comprising primary tumors and lymph node metastases was analyzed. Overall 52 605 phosphospeptides, including mono‐ and multiply phosphorylated peptides were identified in 6939 proteins (Figures 5A‐5B and Table S4). These proteins were matched to 6793 unique coding genes. Interestingly, this melanoma phosphoproteome contributed with 470 additional proteins to the melanoma proteome reached through global proteomics experiments. The melanoma phosphoproteome is distributed throughout the whole protein abundance range. Moreover, a fraction of the phosphoproteome correspond to very low‐abundant proteins that were only detected after phosphopeptide enrichment (Figure 5B). Besides, the mapped phosphoproteome is widely distributed across most of the cellular pathways and processes, including all described signaling pathways dysregulated during melanoma development and progression (Figure 5C). Particulary for the MAPK phosphorylation signaling cascade, the phosphorylation sites in the majority of intermediates and effector proteins were found.

#### 2.6.2 Melanoma kinome

Protein kinases are essential executors of phosphorylation events in signal transmission, and their comprehensive analysis can offer significant understandings of biological mechanisms. Altered expression or activity of kinases is often involved in disease processes such as immunodeficiencies, endocrine disorders, and cancers. Consequently, protein kinases have been extensively studied to identify drug targets for therapy, define new biomarkers, or discover drug efficiency related biomarkers.

The melanoma kinome was described based on computational kinase‐specific phosphorylation site prediction from the phosphoproteome data and direct proteomic kinase identification. We found 38 392 phosphopeptides linked to 210 phosphorylation motifs (Figure 5D and Table S5), which translated into the prediction for 244 kinases (Table S6). As an example, MAPK3 and MAPK1 (ERK1 and ERK2), two important kinases known to be involved in melanoma development and progression, were predicted based on the identification of 695 and 1408 phosphorylated peptides respectively. In total, the phosphorylated peptides were mapped to 65 different substrate proteins highlighting the fact that most of these proteins are targeted by ERK1/2 in multiple sites. The protein interaction network of the identified ERK1/2 substrates reveals that a large subset of these proteins is already reported as ERK1/2 targets. Moreover, the functional annotation enrichment analysis exposes a role of ERK1/2 in the regulation of critical signaling cascades for cancer cells such as the MAPK, ErbB, mTOR, HIF‐1, and PI3K‐Akt pathways, and also in the regulation of the actin cytoskeleton (Figure S3). On the other hand, 425 kinases were directly identified in the melanoma proteome data generated (Figure 5E). Overall, the melanoma kinome data covered more than 84% (522) of the defined human kinome. Identified and predicted kinases were displayed in a dynamic force‐directed kinome network using Coral,^78^ encoding qualitative kinase attributes in branch and node colors. The kinases were rather evenly distributed across all major classes of this protein family (Figure 5F, Table S7).

#### 2.6.3 Lysine acetylome

The lysine acetylome was analyzed for 60 melanoma tumor samples including primary tumors, and metastases. For the identification of site‐specific acetylated proteins full chemical acetylation of free amino groups followed by trypsin digestion of the modified proteins was performed. Generated peptides were delimited by arginine residues because trypsin cannot cleave after acetyl‐lysine residues, thus resembling the results of Arg‐C‐like digestion. Chemically incorporated acetyl groups carried heavy isotopes to differentiate them from endogenous acetylation. This strategy allows not only the identification of site‐specific lysine acetylation sites but also the quantification of their occupancy.^79^, ^80^

**FIGURE 6:**
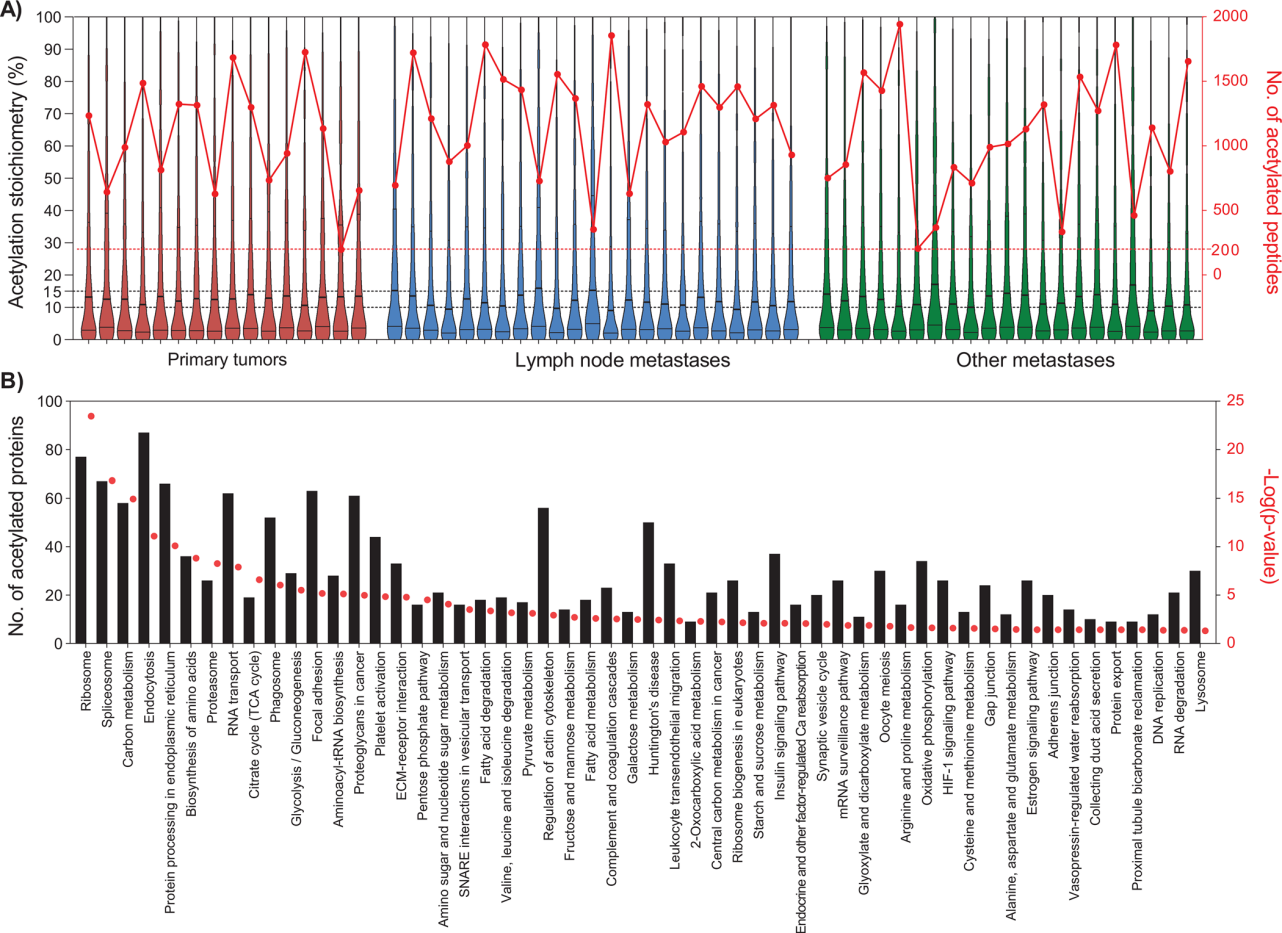
Distribution of the acetylome identified in melanoma tumors. (A) Violin plots showing the distribution of the site‐specific acetylation occupancy (acetylation stoichiometry [%], left axis) of peptides in the 60 samples submitted to acetylome analysis. The samples were grouped according to their origin: primary tumors (red), lymph node (blue), and other metastases (green). The number of acetylated peptides identified in each sample is represented with red dots and connected lines within origin based groups (no. of acetylated peptides, right axis). (B) KEGG pathways significantly enriched in the melanoma acetylome. Bars correspond to the number of acetylated proteins involved in the annotated pathway. The enrichment –log(*P* value) represented as red dots was plotted for each pathway annotation (right axis)

Among the analyzed samples, 16 correspond to primary melanoma, 23 to lymph node metastases, and 21 to metastases found in other organs. The results did not show major differences in terms of identification of acetylated peptides or the distribution of their site‐specific occupancy (Figure 6A). The number of acetylated peptides by samples ranged from 200 to 2000, which depended on the total number of identified peptides (Figure 6A). Despite of a wide range of identified peptides, the distribution of the acetylation occupancy, represented as violin plots, were very similar across all samples under study. In total we identified 4421 acetylated peptides corresponding to 2325 proteins (Table S1). The abundance distribution of acetylated proteins showed a shift toward the high abundant proteins (Figure 5B), which is linked to a technical limitation of current MS instruments and the fact that the vast majority of acetylation sites show low occupancy (Figure 6A).

On average, the acetylation site occupancy was below 15% in the majority of the samples. These findings are in agreement with previous results reported by our group and others.^79^, ^80^, ^81^, ^82^ Metabolic pathways such as glycolysis, the TCA cycle, and amino acid and fatty acid metabolism, were significantly enriched in the melanoma acetylome (Figure 6B). Coincidently, these pathways have been found dysregulated in melanoma with important implications to the progression of the disease^83^ (Figure 6B). Previous reports have also pointed at pathways and proteins regulated by acetylation, which were also found enriched in our melanoma acetylome. These included ribosomes, proteins involved in the translation machinery, transcription, and RNA processing at different levels.^83^, ^84^ Furthermore, our differential analysis between transcriptomics and proteomics revealed a disparate enrichment for most of these pathways (Figure 3C), which might indicate a potential role for acetylation in the stability of target proteins.

Our findings confirm that lysine acetylation is a widespread PTM and regulate an increasing number of biological pathways and processes. The melanoma acetylome provides the foundation to better understand the regulatory mechanisms driven by acetylation and controlling enzymes, and to explore new therapeutic opportunities.

Both phosphorylation and acetylation regulate a large and increasing number of proteins with known implications in the pathogenesis of melanoma.

### 2.7 Drug therapy directed signatures of protein expression

**FIGURE 7:**
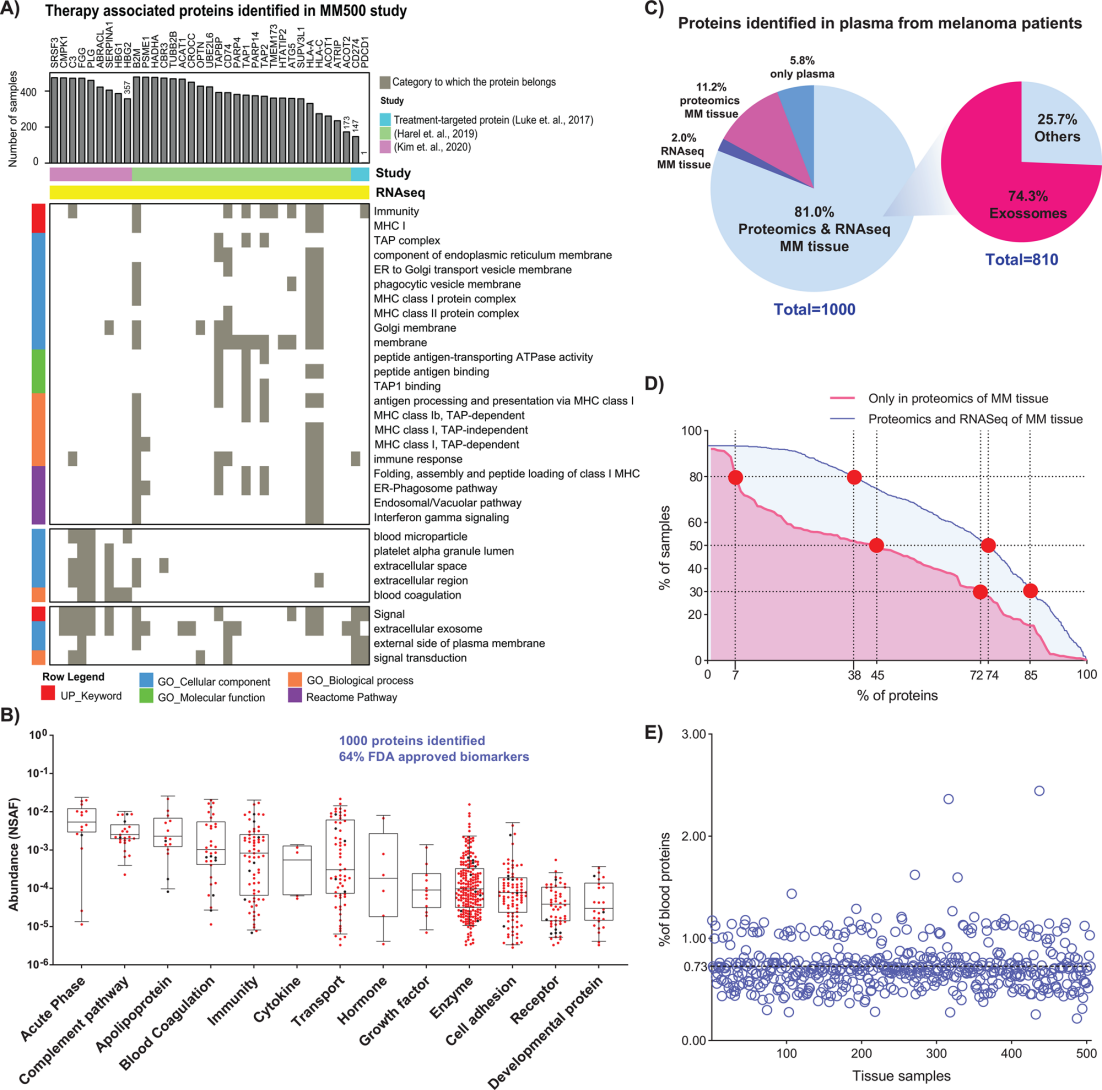
Melanoma therapy‐associated proteins and blood plasma protein profiles from pooled patient samples. (A) Distribution of 35 therapy‐associated proteins identified in our samples (Top panel).The bar length indicates the number of MM500 tumor samples where the proteins were identified. The bottom panel shows a functional clustering of these proteins. The analysis was performed including nine proteins previously described by our lab as responders to several drug treatments, two well‐known treatment‐targeted proteins (CD274, PDCD1), and a signature of 24 proteins described by Harel et al (2019) as markers of response to immunotherapy.^80^ (B)  Box‐plot of quantified proteins in plasma, related to the protein classes, and functions. The abundances were calculated according to NSAF criteria. (C) Pie chart representation of the 1000 proteins identified in an a pool of blood plasma of melanoma patients. The figure single out specific fractions that have been identified in proteomic or transcriptomic studies on MM tissues as well as those related to exosomal expression. (D) Representation of plasma proteins distribution among tissue samples. The *x*‐axis represent the percentage of plasma proteins categorized as proteins originating from blood plasma (in red “only in Proteomics of MM tissues”) or proteins identified in blood plasma and also expressed in MM tissues (in blue “Proteomics and RNASeq of MM tissues”). The *y*‐axis represents the percentage of MM tissue samples where the plasma proteins were identified. The intersection points marked in red represent the percentage of samples (30%, 50%, and 80%) where the plasma proteins were identified. (E) Distribution of protein originating from blood plasma across MM tissue samples. The *x*‐axis represents the tissue samples and *y*‐axis represents the percentage of proteins originating from blood plasma that were identified in MM samples (100 × (# proteins originating from blood/total number of proteins in MM tissue))

Protein profiling studies that involve mass spectrometry‐based proteomics have been utilized to analyse and evaluate the regulation of proteins under various conditions including therapy, elucidate molecular mechanisms, and determine the status of protein networks in melanoma.^85^, ^86^ In the MM500 study we identified 35 proteins recognized as dysregulated in tumors of melanoma patients under different treatment schemes.^86^, ^87^, ^88^ All these proteins have also been detected at the transcription level (according to the TGCA repository) in melanoma tumor (Figure 7A, top panel, yellow colour [RNAseq]). A functional annotation clustering performed with these proteins (https://david.ncifcrf.gov/home.jsp), revealed three major functional clusters related to immunity, extracellular activities and signalling respectively (Figure 7A, bottom panel). Nine of these proteins, which were previously reported by our group as also associated with melanoma treatment^88^ were here identified in more than 350 (> 70%) samples (Figure 7A, top panel, indicated in magenta colour). Notably, the proteins SRSF3, PLG, FGG, C3, and SERPINA1 have also been related to survival in melanoma patients.^68^ According to the functional annotation clustering, these proteins are mostly extracellular and associated with cell signalling in the case of C3, with immune response (Figure 7A, bottom panel). Two of the main treatment approaches used in melanoma include strategies to target the CTLA‐4 protein and Programmed Death‐1/Programmed Death Ligand‐1 (PD‐1/PD‐L1). In our data, PD‐L1 (CD247) was successfully identified in 147 melanoma samples (Figure 7A, top panel). However, the protein PD‐1 (PDCD1) was only identified in one sample and we were unable to identify CTLA‐4, despite of the large number of samples studied and LC‐MS/MS experiments. According to the Peptide Atlas (http://www.peptideatlas.org, “*24 November 2020, date last accessed*”),^89^ these two proteins have only been reported once in independent mass spectrometry studies.^90^, ^91^ Consequently, we followed the 24 proteins identified by Harel et al^86^ as proteomics signatures of the melanoma response to immunotherapy (Figure 7A, top panel, indicated in green colour). These proteins were detected in 173 samples of our study, and a fraction of them (19) were identified in 357 samples. The functional annotation clustering revealed that they are related to immune response, interferon‐gamma signalling, MHC 1 and 2 complexes, among others (Figure 7A, bottom panel).

**FIGURE 8:**
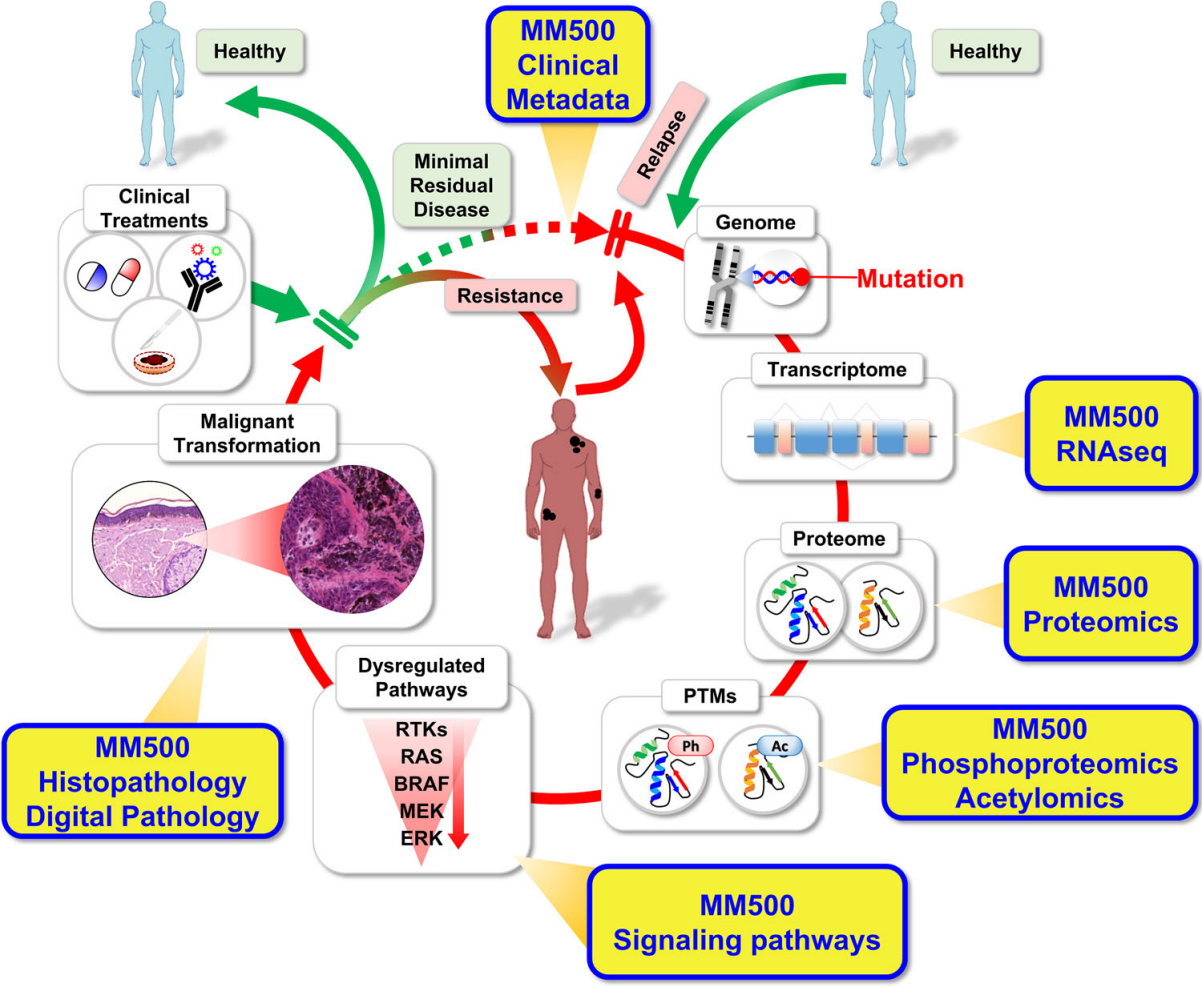
The Proposed impact of the MM500 study data under the clinical treatment cycle, where the patient from a healthy state enters into a progressive disease evolvement process. The first indication of early melanoma disease onset is discovered by an image capture, for example, CT (high resolution), and/or MR, accompanied by a genomic mutation signature, along with protein localization and expression. Assignments of posttranslational modifications, as well as pathway biology activations, and histopathology characterizations added to the disease status. Based on the diagnosis output, a dedicated and optimized drug treatment is presented to the patient. The melanoma disease cycle is reinforced upon metastasis development, that entails a resistance build up

### 2.8 Protein expression signature in pooled plasma

Blood sampling and automated fractionation into plasma, serum, lymphocytes, and erythrocytes, were conducted within the study. Fifteen percent of the entire sample set was mapped in pooled plasma. Within this sample set, approx. 8505 peptide sequences were annotated, resulting in more than 1000 identified proteins (Table S8). These results constitute the first plasma proteome profile of melanoma patients, performed by our pooling principle. The plasma proteins identified were widely distributed according to their class and cellular function (Figure 7B). By relating to all FDA‐approved plasma biomarkers, the present data verified 63% of these disease markers.^92^

Most of the proteins identified in plasma were detected at both the transcript and protein levels in the melanoma tumors (Figure 7C). We hypothesize that proteins originating from blood plasma were not detected in RNASeq experiments of tissue samples. These proteins (112 in total) were identified with lower frequency in tissue samples than the rest of the proteins (Figure 7D and Table S8). For example, 45% of the proteins originating from blood plasma were present in 252 (approx. 50%) of the tissue samples whereas, in this same number of samples were detected 74% of the plasma proteins with transcript evidence. We found that less than 3% of the proteins identified in each melanoma tissue sample originated from blood plasma, and for 85% of the samples, these proteins represented less than 1% of all the identifications (Figure 7E). Overall, 84% of the proteins originating from blood plasma identified in the analysis of tumor samples were immunoglobulins. Though sample preparation may have influenced the crossed identification of plasma proteins in tissue samples, other factors including the vascularization and immune components should be considered, as they reflect important aspects of tumor development in interaction with the microenvironment.

Interestingly, the proteins identified by both transcriptomics and proteomics of melanoma tumors have previously been identified in exosomes^93^ (http://www.exocarta.org/).^94^ Thus, a major part of the proteins annotated in the plasma samples may be of exosome origin. The exosomes are membrane‐bound extracellular vesicles of endothelial origin, and there is a growing interest for exosomes as potential clinical use as biomarkers. Despite emerging evidence of bioactive material transport by exosomes in melanoma, the functions of exosomes in cancer progression remains fundamentally unknown.^95^, ^96^, ^97^

## 3 CONCLUSIONS

By analyzing a wide range of well‐characterized primary and metastatic tumors, a “Melanoma Protein Blueprint” was built. In comparison to the recent publication “High‐Stringency Blueprint of the Human Proteome,”^1^ covering 90.4% of the human protein‐coding genes, the current study of the melanoma proteome has an overlap of approximately 74% with the observed human proteome.

A database was established that covers proteins that can be expected in any melanoma, whether these are primary or metastatic and could be used in further research for the identification of prognostic and predictive factors in melanoma. The potential impact of the present dataset under the clinical treatment cycle of a typical melanoma patient is visualized in Figure 8.

With the 15 973 expressed proteins within the Melanoma Proteome, a remarkably high fraction is dedicated to cell division, splicing mechanisms, and metabolism, highlighting the biological challenge of maintaining normal protein function and structure in cancer. One finding suggests that the mitochondrial function is of particular importance in melanoma.^98^, ^99^, ^100^

Our study also indicated that several mutations associated with genomic classification and drivers of the disease, are expressed at very low levels in melanoma tumors. Hence, approaches such as targeted proteomics in combination with peptide fractionation or enrichment protocols should be used to overcome this barrier, and successfully identify and quantify more of the key mutations at the protein level in melanoma.

In summary, a comprehensive analysis of the overall proteome in melanoma is presented, based on well‐annotated tumor tissues combined with blood samples. This molecular signature data in combination with the in‐depth histopathological characterization of the tumors may support the discovery and development of key biomarkers as well as new drug development.

## 4 MATERIALS AND METHODS

Note: Chemicals, reagents, tissue specimens, sample preparation for mass spectrometry, LC‐MS/MS analysis and protein identification were similar, and performed as described in the manuscript “The Human Melanoma Proteome Atlas—Defining the Molecular Pathology Expression,” submitted to this same journal. In addition, several methods were additionally introduced in the present manuscript: Immunodepletion of the 14 most abundant proteins from plasma; automated, phosphopeptide enrichment by Fe(III)‐IMAC‐based workflow; data analysis for melanoma phosphoproteome, acetylome, and kinome.

### 4.1 Chemicals and reagents

Dithiothreitol (DTT), iodoacetamide, ammonium bicarbonate (Ambic), ammonium hydroxide, sodium docecylsulphate (SDS), trifluoroacetic acid (TFA), sodium deoxycholate (SDC), tris(hydroxymethyl)aminomethane (Tris), formic‐acid, and urea were purchased from Sigma‐Aldrich (St. Louis, MO, USA). Triethylamonium bicarbonate (TEAB) and hydroxylamine were from Thermo Fisher Scientific. Water and organic solvents were all LC‐MS grade and supplied by Merck (Darmstadt, Germany) or (Thermo Fisher Scientific). Endoproteinase Lys‐C was obtained from Wako (Osaka, Japan) and sequencing‐grade modified trypsin was purchased from Promega (Madison, WI, USA). Cell lines SK MEL2 (HTB‐68), SK MEL28 (HTB‐72), and RPMI‐7951 (HTB‐66) were obtained from the American Type Culture Collection (ATCC).

### 4.2 Tissue specimen

Tumor samples were obtained from University clinics in Sweden and Hungary (Table S1A‐B of the manuscript “The Human Melanoma Proteome Atlas—Defining the Molecular Pathology Expression”). All studies were approved by the local ethical committees; at Lund University, Southern Sweden (DNR 191/2007, BioMEL biobank 101/2013, 2015/266 and 2015/618), at Semmelweis University, Hungary (191‐4/2014), and University of Szeged, Hungary (MEL‐PROTEO‐001). All patients provided written informed consent. The malignant melanoma primary and metastatic tissue samples were snap‐frozen within 30 minutes after surgical resection with a small amount of isopentane in liquid nitrogen or put on dry ice within 20 minutes of collection. Multiple pieces were collected from most of the tumor specimens. The source of the analyzed tissue samples and patients who provided them was as follows: Lund University Hospital 289 samples from 147 patients, Semmelweis University Hospital 165 samples from 75 patients, and Szeged University Hospital 51 samples from 10 patients.

Fresh‐frozen tissues collected at Lund University Hospital were stored in the Melanoma biobank, BioMEL, Region Skåne, Sweden. Tissues collected at sites in Hungary were stored at the respective biobanks of Semmelweis University and the University of Szeged. Samples transportation from Hungary was carried out in liquid nitrogen for the tissues or put on dry ice for the blood samples. In the case of formalin‐fixed paraffin‐embedded (FFPE) samples, the fixation of tumor tissues was performed right after surgery with 4% cc. buffered formaldehyde. Samples were then dehydrated in xylene/alcohol solution and embedded into paraffin and stored at room temperature. Sections of 10 μm were used for further analysis. The study has been performed in compliance with GDPR.

All tumors were processed with integrated Biobanking consolidations within all involved medical centers. The workflow was built according to Swedish biobanking laws and best practices and guidelines provided by the BBMRI‐ERIC, ESBB, and ISBER (https://www.bbmri‐eric.eu/services/quality‐management).^101^ The process flow enabled rapid sample handling whereby collected tissues were stored at an ultra‐low temperature in a biobank at a cycle time of approx. 20 minutes. Using the same data management system and database reconnaissance, sample integrity was ensured via electronic surveillance. The patient and sample processing workflow and protocols were transferred and integrated at all of the clinical centers and interfaced with the RedCap database (https://www.project‐redcap.org/).^102^

### 4.3 Cell cultures

For cell culture, melanoma cell lines (SK‐MEL‐2, SKMEL28, and VMM1) were purchased from American type culture collection (ATCC). All cell lines were cultured and maintained in the standard conditions and recommendations according to the manufacturer's instructions. In detail, SK‐MEL‐2 and SKMEL28 were maintained with DMEM (Dulbecco's modified Eagle's medium) supplemented with 10% fetal bovine serum (FBS) and penicillin‐streptomycin (P/S).VMM1 were maintained with RPMI‐1640 supplemented with 10% FBS and P/S. All cells were maintained at 37°C in a humidified 5% CO_2_ incubator.

### 4.4 Plasma samples

Blood samples from 47 melanoma patients from Semmelweis University Hospital, whose tumors were also included in the MM500 study were subjected to automated fractionation into plasma, serum, lymphocytes, and erythrocytes as published before.^103^, ^104^ For some patients several samples were taken at a different stage of the disease and a pool of 57 plasma samples was prepared.

### 4.5 Histopathological analysis

Stepwise sectioning of the tissues was performed, and on average, three sections were evaluated. Tissue sections were placed on glass slides, stained with hematoxylin and eosin, and then placed in an automated slide scanner system (Zeiss Mirax, Jena, Germany). The slides were then evaluated for tissue content: tumor, necrosis, connective tissue, and adjacent background tissue—mostly lymphatic cells—lymph node area.^68^

### 4.6 Sample preparation for mass spectrometry

#### 4.6.1 Deparaffinization of FFPE tissue

The FFPE tissue sections were incubated with 1 mL of 1:50 diluted EnVision™ FLEX Target Retrieval Solution High pH (Agilent Dako) at 97˚C for 10 minutes (500 RPM). Incubation was followed by a brief centrifugation at 14 000 *g* at 4˚C for 3 minutes, removal of the EnVision solution and the paraffin. These steps were repeated until complete paraffin removal as previously described.^81^

#### 4.6.2 Immunodepletion of the 14 most abundant proteins from plasma

A pool of 57 plasma samples from 47 melanoma patients was submitted to immunodepletion using a Multiaffinity Removal Column human‐14 (4,6 × 100 mm) (Agilent Technologies), according to the instructions provides by the manufacturer. Buffer exchange using Amicon Ultra Centrifugal filter (0.5 mL—10 kDa, Millipore, Ireland) was performed following depletion. After being transferred to the Amicon 10 kDa, the samples were centrifuged at 13 000 *g* for 20 minutes. One more step of centrifugation was done at 13 000 *g* for 20 minutes, following the addition of 400 μL 50 mM Ambic. The last step was repeated, and the samples were centrifuged for 30 minutes. Finally, 70 μL of 10% SDS/25 mM DTT in 100 mM TEAB was transferred to the Amicon 10 kDa and the sample recovering was performed by centrifugation at 1000 *g* for 5 minutes.

#### 4.6.3 Protein extraction

For fresh‐frozen tissues, the lysis buffers contained 100 mM ammonium bicarbonate or 100 mM Tris pH 8.6 and up to 6 M Urea or 2% SDS. Lysates were generated by sonication in an ice bath using a Branson Sonifier 250 (output 4, 10% duty cycle) or using the Bioruptor plus, model UCD‐300 (Dieagenode) for 40 cycles. Each cycle consisted of 15 seconds at high power and 15 seconds without sonication at 4°C. Samples were centrifuged at 10 000 *g* and 4°C for 10 minutes and the supernatants were transferred into a new tube and the pellet was discarded.

In the case of FFPE tissue samples, the protein extraction was performed by adding 100 mM TEAB containing 25 mM DTT and 10w/v% SDS pH 8. The samples were incubated at 99˚C for 1 hour with shaking (500 RPM) and sonicated in the Bioruptor® Plus UCD‐300 (Diagenode) for 40 cycles (15 seconds on and 15 seconds off) at 4˚C, followed by centrifugation at 20 000 *g* at 18˚C for 10 minutes.

#### 4.6.4 Protein determination

The protein in each one of the samples was determined using a colorimetric micro BCA Protein Assay kit (Thermo Fisher Scientific, Rockford, IL) following the manufacturer's instructions.

#### 4.6.5 Protein digestion

Proteins were reduced with 10 mM DTT for 1 hour at 37°C and alkylated with 40 or 50 mM iodoacetamide for 30 minutes, in the dark, at room temperature. Proteins were digested overnight with trypsin or Lys‐C and trypsin using published and optimized protocols including buffer exchange^68^, ^105^, ^106^ or urea in‐solution digestion^107^ which comprised automated sample handling.^108^ SDS was removed from the samples by the MED‐FASP^109^ method or by ethanol precipitation.^80^ The later was followed by protein solubilization in 50 mM Ambic with 0.5 SDC (Sodium deoxycholate) and trypsin digestion. For acetylation analysis, the samples were processed and digested as previously described which resembles an Arg‐C like enzymatic hydrolysis^80^ (see Material and Methods of Supporting Information).

FFPE‐derived protein extracts were digested using the S‐trap method following the manufactures’ instructions with a few modifications as reported in recently.^81^

The undepleted plasma sample was diluted in Miliq water in a ratio of 1:10. Approximately 70 μg of protein of each sample was added to 42.25 μL of 10% SDS/25 mM DTT in 100 mM TEAB solution before protein digestion. Undepleted and depleted plasma samples were then incubated for 5 minutes at 99 °C, 500 rpm. Samples were alkylated with iodoacetamide in a final concentration of 50 mM for 30 minutes in the dark, at room temperature. Protein digestion was carried out also using the S‐trap methodology.

#### 4.6.6 Automated Fe(III)‐IMAC‐based workflow

We enriched phosphorylated peptides using the Phospho Enrichment v2.0 protocol on the AssayMAP Bravo platform as previously described.^110^ The 5 μL Fe(III)‐NTA cartridges were primed with 100 μL 50% ACN, 0.1% TFA at a flow rate of 300 μL/min and equilibrated with 50 μL loading buffer (80% ACN, 0.1% TFA) at 10 μL/min. High pH Rp‐fractions obtained previously were loaded onto the cartridge at 3.5 μL/min. The columns were washed with 50 μL loading buffer and the elution of phosphorylated peptides was performed with 25 μL 5% ammonia directly into 10 μL 50% formic acid (FA). Samples were lyophilized in a vacuum concentrator and stored at –80°C until analysis by LC‐MS/MS.

#### 4.6.7 TMT 11 plex labeling

TMT labeling was performed according to manufacturer's instructions.

#### 4.6.8 Peptide fractionation

TMT‐11 and labeled‐free peptides were separated by basic pH reversed‐phase liquid chromatography (HpH RP‐HPLC) on a Phenomenex Aeris C8 column (100 mm  ×  2.1 mm, 3.6‐μm particles) using an Agilent 1100 HPLC system and a gradient with solvent A 20 mM ammonium formate (pH 10) and solvent B 80% ACN‐20% water containing 20 mM ammonium formate (pH 10). Labeled‐free peptides were also fractionated by strong cation exchange (SCX) using Microspin columns (MA SEM HIL‐SCX, 10‐100 μg capacity, The Nest group Inc., South Borough) in stepwise elution.^105^, ^106^

#### 4.6.9 Peptide desalting

Enzymatic digestions were quenched by adding formic acid to a final concentration of 1%. Proteolytic peptides were desalted prior to LC‐MS/MS experiments. We used C18‐microcolumns (The Nest Group, MA, USA) following the manufacturer's instruction, or the AssayMAP Bravo platform using the peptide cleanup v2.0 protocol with C18 cartridges (Agilent, 5 μL bed volume). Peptides were eluted in 80% ACN, 0.1% TFA, dried on a Speevac, and dissolved in 0.1% formic acid or 0.1% TFA. Peptides generated by digestion with SDC protocol or on the S‐traps were directly analyzed by LC‐MS/MS without desalting.

#### 4.6.10 Peptide determination

The peptide quantity in each sample and fraction was determined using the Pierce Quantitative Colorimetric Peptide Assay according to the instructions provided by the manufacturer.

### 4.7 LC‐MS/MS analysis

We used two main LC‐MS/MS setups: System 1 and System 2. System 1 comprised an Easy nLC‐1000 (Thermo Fisher Scientific) coupled to a Q Exactive Plus mass spectrometer (Thermo Fisher Scientific, San Jose, CA). Here the peptides (∼1 μg) were initially loaded onto a trap column (Acclaim PepMap 100 precolumn, 75 μm i.d. × 2 cm, C18, 3 μm, 100 Å; ThermoFisher Scientific, San Jose, CA) and then separated on an analytical column (EASY‐Spray column, 75 μm i.d. × 25 cm, PepMap RSLC C18, 2 μm, 100 Å; ThermoFisher Scientific, San Jose, CA). System 2 comprised an Ultimate 3000 nLC (Thermo Scientific, San José, CA, USA, Bremen Germany) coupled to a Q Exactive HF‐X mass spectrometer (Thermo Scientific). For this case, the peptides (∼1 μg) were loaded in a trap column (Acclaim1 PepMap 100 precolumn, 75 μm, 2 cm, C18, 3 μm, 100 Å, Thermo Scientific) and then separated on an analytical column (EASY‐Spray column 25 or 50 cm, 75 μm i.d., PepMap RSLC C18, 2 μm, 100Å, Thermo Scientific). Both systems used a flow rate of 300 nL/min and a water/ACN gradient in 0.1% formic acid and samples were measured in DDA and DIA modes. The DIA‐MS Spectral library was built out of DDA‐LC‐MS/MS analyses of samples from tissue and cultured cell origin, with spiked in iRT peptides (Biognosis AG). This also included the analysis of the mixture of samples previously fractionated by HpH RP‐HPLC.

### 4.8 Data analysis

#### 4.8.1 Peptide and protein identification and quantitation in DDA‐MS experiments

Raw DDA‐LC‐MS/MS files were analyzed with the Proteome Discoverer™ Software (Thermo Scientific™) against Uniprot Human dataset to which were added Fasta format protein sequences of known driver mutations of melanoma disease.^35^ The search engine Sequest HT was used for peptide identification. Carbamidomethylation was set as a static modification as well as TMT 6 plex (+229.1629 Da) at peptide N‐terminus and lysine for labeling experiments. Oxidation of methionine residues and acetylation at protein N‐termini were selected as dynamic modifications. Precursor and fragment mass tolerance was set as 20 ppm and 0.02 Da, respectively, and two missed cleavages were allowed for peptides. For the case of phosphopeptides, the ptmRS algorithm was used to score phosphorylation sites with a site probability threshold >75. The Minora node was included in the search for identification using retention time alignment and the match‐between‐runs features. For label‐free experiments the quantification was carried out using the TOP3 method where the protein abundance is reported as the mean of the three highest peptides (unique and razor) areas measured for each protein. For TMT labelling experiments protein abundances were calculated as the summed areas of reporter ions considering unique peptides. Identification and sorting of unique peptides of missing proteins were carried using the neXtProt tool “Peptide uniqueness checker” (https://www.nextprot.org/tools/peptide‐uniqueness‐checker).^111^

#### 4.8.2 Peptide and protein identification and quantitation in DIA‐MS experiments

A Global proteomics spectral library was generated from DDA experiments as described above. Raw files were converted to HTRMS files with a special converter provided by Biognosys AG and searched in the Spectronaut X platform (Biognosis AG) against the Homo sapiens database from Uniprot containing isoforms. Dynamic retention time prediction was selected to enable nonlinear alignment of precursor retention times between the (iRT, normalized retention time) spectral library and the DIA‐MS data by segmented regression. The following parameters were used: cysteine carbamidomethylation (+57.0215 Da) as fixed modification and methionine oxidation (+15.9949 Da), N‐terminal acetylation (+42.0105 Da) as dynamic modifications. A maximum of two missed cleavages were accepted. Precursor mass tolerance was set to 10 ppm and for the MS/MS fragments it was set to 0.02 Da. Between 3 and 25 fragments were collected per peptide. Phosphorylation (+79.9663 Da) on serine, threonine, and tyrosine was selected as variable modifications for the phosphoproteomics analysis. The phosphosite localization algorithm was set according to previous description.^112^ Phosphosites with a score that was equal or higher than 0.75 were considered as Class I. Filtering was performed at a 1% false discovery rate (FDR) for all the peptides and proteins that were used to construct the spectral library. The resulted library containing identified spectra for 220 360 peptides representing 12 293 proteins. The software computed MS1 peptide abundance as the summed precursor XIC (Extracted‐Ion Chromatogram, from the monoisotopic precursor ion plus isotopic envelope). The protein abundance resulted from the average of the top three most intense precursor ions corresponding to unique and razor peptides.

### 4.9 Bioinformatic and statistical analysis

#### 4.9.1 Protein normalization

The results from protein identification and quantification were imported into Perseus software.^113^ Data were normalized by log2 transforming the protein intensities, and standardization was performed by subtracting individual values by the median in each sample. The proteins showing less variability across all batches that were identified in 100% of the samples were used to correct the abundance differences between batches. To do that, individual protein intensities in each batch were subtracted by the median abundance of selected proteins in the specific batch. After correction, the median abundance for each protein across all samples was calculated and reported as the relative abundance in our melanoma proteome.

#### 4.9.2 Stoichiometry of acetylated lysines

The lysine acetylation stoichiometry identification and quantification were estimated as previously described.^80^, ^81^ Briefly, raw files were analyzed with Pview software to identify and calculate the site‐specific acetylation occupancy. Also, only those peptides identified in both, Pview and Proteome Discoverer were considered for reporting their acetylation stoichiometry.

#### 4.9.3 Kinase‐specific phosphorylation site prediction

Phosphopeptides sequences were edited to include “#” in front of the S, T, or Y phosphorylation sites. The background database consisted of a fasta file from all identified phosphorylated proteins in this study. The software motifeR^112^ was used to align the phosphopeptide sequences with the background database, providing a uniform sequence length of 15 amino acids. The motifeR was also used to enrich phosphorylation motifs and retrieve kinase‐substrate annotation. All kinases identified in the MM500 proteome and kinases predicted by the enriched motifs were visualized in the context of the human kinome superfamily using Coral.^78^

## DATA AND CODE AVAILABILITY

The data that support the findings of this study are openly available in ProteomeXchange at http://www.proteomexchange.org/, reference numbers PXD001725, PXD001724, PXD009630, PXD017968, and PXD026086 and will be complemented by the addition of more data from the study. The TCGA data was downloaded from cBioPortal https://www.cbioportal.org. The code for MM500 study can be found at https://github.com/rhong3/TCGA_melanoma. Table S1 of Supporting Information is available at https://github.com/rhong3/TCGA_melanoma/tree/master/Supporting%20Information%20tables.

## Supporting information

SUPPORTING INFORMATION

SUPPORTING INFORMATION

SUPPORTING INFORMATION

SUPPORTING INFORMATION

SUPPORTING INFORMATION
