# Supplementary material for: The Human Melanoma Proteome Atlas—Complementing the melanoma transcriptome
Source: Clin Transl Med. 2021 Jul 22;11(7):e451. doi: 10.1002/ctm2.451 (PMC8299047; doi:10.1002/ctm2.451)
Supplement: Supplementary file 1 — SUPPORTING INFORMATION [file CTM2-11-e451-s002.docx]

**SUPPORTING INFORMATION**

The Human Melanoma Proteome Atlas – Complementing the Melanoma Transcriptome

Lazaro Hiram Betancourt^1*^, Jeovanis Gil^1*^, Aniel Sanchez^2^, Viktória Doma^3,4^, Magdalena Kuras^2^, Jimmy Rodriguez Murillo^5^, Erika Velasquez^2^, Uğur Çakır^6^, Yonghyo Kim^1^, Yutaka Sugihara^1^, Indira Pla Parada^2^, Beáta Seitz^6^, Roger Appelqvist^1^, Elisabet Wieslander^1^, Charlotte Welinder^1^, Natália Pinto de Almeida^7,8^, Nicole Woldmar^7,8^, Matilda Marko-Varga^1^, Jonatan Eriksson^1^, Krzysztof Pawłowski^4,9,10^, Bo Baldetorp^1^, Christian Ingvar^11,12^, Håkan Olsson^1,11^, Lotta Lundgren^1,11^, Henrik Lindberg^1^, Henriett Oskolas^1^, Boram Lee^1^, Ethan Berge^1^, Marie Sjögren^1^, Carina Eriksson^1^, Dasol Kim^13^, Ho Jeong Kwon^13^, Beatrice Knudsen^14^, Melinda Rezeli^8^, Johan Malm^2^, Runyu Hong^15^, Peter Horvath^16^, A. Marcell Szász^17,18^, József Tímár^3^, Sarolta Kárpáti^4^, Peter Horvatovich^19^, Tasso Miliotis^20^, Toshihide Nishimura^21^, Harubumi Kato^22^, Erik Steinfelder^23^, Madalina Oppermann^23^, Ken Miller^23^, Francesco Florindi^24^, Quimin Zhou^25^, Gilberto B. Domont^7^, Luiciana Pizzatti^7^, Fábio C. S. Nogueira^7^, Leticia Szadai^26^, István Balázs Németh^26^, Henrik Ekedahl^1,11^, David Fenyö^15^ and György Marko-Varga^8,13,22^

1. **INSTITUTIONAL AFFILIATIONS**
2. Division of Oncology, Department of Clinical Sciences Lund, Lund University, 221 85 Lund, Sweden
3. Section for Clinical Chemistry, Department of Translational Medicine, Lund University, Skåne University Hospital Malmö, 205 02 Malmö, Sweden
4. 2nd Department of Pathology, Semmelweis University, Hungary
5. Department of Dermatology, Venerology and Dermatooncology, Semmelweis University, Hungary
6. Department of Biochemistry and Biophysics, Karolinska Institute, Stockholm, Sweden
7. Department of Internal Medicine and Oncology, Semmelweis University, Budapest, Hungary
8. Chemistry Institute, Federal University of Rio de Janeiro, Rio de Janeiro, Brazil
9. Clinical Protein Science & Imaging, Biomedical Centre, Department of Biomedical Engineering, Lund University, BMC D13, 221 84 Lund, Sweden
10. Department of Molecular Biology, University of Texas Southwestern medical center, Texas, USA
11. Department of Biochemistry and Microbiology, Warsaw University of Life Sciences, Warszawa, Poland
12. SUS University hospital Lund, Lund, Sweden
13. Department of Surgery, Clinical Sciences, Lund University, SUS, Lund, Sweden
14. Chemical Genomics Global Research Lab, Department of Biotechnology, College of Life Science and Biotechnology, Yonsei University, Seoul, Republic of Korea
15. Department of Pathology, University of Utah, Salt Lake City, USA
16. Department of Biochemistry and Molecular Pharmacology, Institute for Systems Genetics, New York University Grossman School of Medicine, New York, USA
17. Synthetic and Systems Biology Unit, Biological Research Center, 6726 Szeged, Hungary
18. Department of Bioinformatics, Semmelweis University, Budapest, Hungary
19. Department of Internal Medicine and Oncology, Semmelweis University, Budapest, Hungary
20. Department of Analytical Biochemistry, Faculty of Science and Engineering, University of Groningen, Groningen, The Netherlands
21. Translational Science and Experimental Medicine, Cardiovascular, Renal and Metabolism, IMED Biotech Unit, AstraZeneca, Gothenburg, Sweden
22. Department of Oncology, St. Marianna University School of Medicine, Kanagawa, Japan
23. 1^st^ Department of Surgery, Tokyo Medical University, Tokyo, Japan
24. ThermoFisher Scientific, HQ, San Jose, CA, USA
25. BBMRI-ERIC HQ, Graz, Austria
26. Department of Plastic and Reconstructive Surgery, Shanghai Ninth People's Hospital, Shanghai Jiao Tong University School of Medicine, Shanghai, China
27. Department of Dermatology and Allergology, University of Szeged, Szeged, Hungary

* These authors contributed equally to this work

Corresponding Author: [lazaro_hiram.betancourt_nunez@med.lu.se](mailto:lazaro_hiram.betancourt_nunez@med.lu.se)

**Table of Content**

| **Supporting Information** | **Legend** | **File name and format** |
| --- | --- | --- |
| Material and Methods | Protein digestion and sample preparation protocols | See below on this doc |
| Table 1 | Proteins identified and quantified in global proteomics, phosphoproteomics and acetylomics in the MM study | The Human Melanoma Proteome Atlas – Complementing the Melanoma Transcriptome _Supporting Information Table 1.zip |
| Table 2 | List of protein-coding-genes identified | Supporting Information Tables 2- 8.xlsx |
| Table 3 | Missing proteins and corresponding unique peptides | Supporting Information Tables 2- 8.xlsx |
| Table 4 | Identification of phosphopeptides and phosphorylation sites | Supporting Information Tables 2- 8.xlsx |
| Table 5 | List of kinase phosphorylation motifs based on phosphopeptide identification | Supporting Information Tables 2- 8.xlsx |
| Table 6 | Predicted kinases, the corresponding substrates and targeted phosphorylation sites | Supporting Information Tables 2- 8.xlsx |
| Table 7 | List of predicted and identified kinases in the MM500 study | Supporting Information Tables 2- 8.xlsx |
| Table 8 | Proteins identified in the pool of plasma samples from melanoma patients | Supporting Information Tables 2- 8.xlsx |
|  |  |  |
| Figure 1 | A) Distribution of the proteins identified in MM500 with no evidence at the transcript level in the RNA sequencing dataset from melanoma tumors (TCGA repository). Proteins were ranked based on their relative abundance and the number of samples where the protein was detected is plotted (right axis). B) Functional annotation enrichment analysis and protein interaction network of identified proteins with no evidence at the transcript level. The functional annotations significantly enriched in the dataset are indicated with different colors (see figure legend). The analysis was performed in STRING and exported to Cytoscape. | Figure-1-Supp Information.eps |
| Figure 2 | Identification of mutations in MM samples. The figure shows the assigned MS/MS spectra of the TMT labeled peptides A) LVVVGAA^12^GVGK corresponding to the protein NRAS with the mutation G12A, B) LVVVGAGD^13^VGK corresponding to the protein KRAS with the mutation G13D, C) LL^114^VDLAEELGHR corresponding to protein CDKN2A with the mutation P114L, D) S^267^SVILFLNK corresponding to the proteins GNA11/GNAQ, which was most likely originated by the mutation N266K, that generated a specific cleavage site for trypsin, at Lys^266^ , E) IGDFGLATE^600^K corresponding to the protein BRAF with the mutation V600E. | Figure-2-Supp Information.tif |
| Figure 3 | Protein interaction network of the predicted ERK1/2 substrates based on the phosphorylation site motifs identified in the MM500 phosphoproteome analysis. Filled in yellow are indicated the kinases MAPK3 (ERK1) and MAPK1 (ERK2), the black connecting lines indicate known interactions with the two kinases, while gray lines highlight the interactions between the identified substrates. The KEGG pathways significantly enriched in the dataset are indicated with different colors (see figure legend). The analysis was performed in STRING and exported to Cytoscape. | Figure-3-Supp Information.eps |

**Material and Methods**

**Protein digestion**

Buffer exchange method

Reduced and alkylated protein samples were subjected to a buffer exchange procedure to remove urea using Amicon Ultra centrifugal filter (0.5mL-10 kDa, Millipore, Ireland). Protein samples were mixed with 200 μL of 50 mM AmBic in the centrifugal filter, centrifuged at 14000 g and 20 °C, for 30 min and the eluates were discarded. These steps were repeated three more times. Samples were transferred to an eppendorff tube and digested overnight with trypsin at a ratio 1:100 w/w (enzyme:protein) ^1,2^.

Digestion using FASP (Filter aided sample preparation) method.

Proteins were processed by the FASP protocol using Microcon 30k centrifugal ultrafiltration units (Merck, Darmstadt) operated at 10000 g. The SDS was removed from the samples by washes (four times) with 100 μL of 50 mM AmBic. Proteins were digested in 50 μL of 50 mM AmBic with trypsin overnight at a ratio 1:100 w/w (enzyme:protein). The released peptides were collected by centrifugation at 10000g for 10 min followed by two washes with 100 μL of 50 mM AmBic.

Multienzymatic digestion (MED) FASP method (MED-FASP).

The samples were processed identically as in the FASP method but the enzymatic digestion was different. Proteins were digested in 50 μL of 50 mM AmBic at 37 °C for 18 h, using Lys-C at an enzyme to protein ratio of 1:100. The released peptides were collected by centrifugation at 10 000 g for 10 min followed by two washes with 100 μL of 50 mM AmBic. The material remaining on the filter was digested in 50 μL of 50 mM AmBic with trypsin at 37 °C for 2 h, at an enzyme to protein ratio of 1:100. This second fraction of peptides was recovered as in the case of the Lys-C digest.

Urea in-solution digestion

Protein digestion was performed on the AssayMAP Bravo (Agilent Technologies) platform using the digestion v2.0 protocol as previously described ^3,4^. Briefly digestions were performed in two steps. Proteins were firstly incubated with Lys-C at a 1:50 (w/w) ratio (enzyme:protein) for 5 h and then trypsin was then added at a 1:50 (w/w) ratio and the mixture incubated overnight at RT.

Digestion on S-trap

The digestion followed the manufacturer's instructions with some variation in the protocol ^5^. Here, 1.2% phosphoric acid was added to the reduced and alkylated protein solutions and S-trap binding buffer (90% methanol,100mM TEAB) to a final volume of eight times the volume of the lysate. The samples were loaded onto the S-trap filter, followed by a short centrifugation. The captured proteins were washed with S-trap binding buffer and centrifuged between each wash. After that, the S-trap column was moved into a new receiver tube/plate. Digestion buffer (50mM TEAB) containing endoproteinase LysC in a 1:50 w/w ratio (enzyme/protein) was added onto the filter and incubated at 37˚C for 2 hours. Then, digestion buffer (50mM TEAB) containing trypsin in a 1:50 w/w ratio (enzyme/protein) was added and the samples were incubated overnight at 37˚C. Next, the peptides were eluted firstly with digestion buffer. The second elution was performed with 0.2% aqueous formic acid, and the final elution consisted of 50% acetonitrile containing 0.2% formic acid. The mixture of eluted peptides was acidified with 50% formic acid to a final pH of ~ 3 and dried down in a centrifugal evaporator.

Lysine acetylation and Arg-C like digestion in SDC

Protein extracts in SDS were digested as previously described ^6^. The proteins were first precipitated with 9 volumes of cold ethanol solution (90%) overnight at −20 °C. The precipitate was solubilized with 0.5% SDS, 0.5% SDC, 0.1 m TEAB, pH 8.0. Acetylation of protein unmodified lysine residues was performed in two consecutive additions of a 100-fold molar excess of deuterated N-Acetoxysuccinimide (NAS-d3) in DMSO and incubated at room temperature for 1 h, respectively. Undesired O-acetylation of serine, threonine and tyrosine residues was reverted by incubating the samples with 5% hydroxylamine for 20 min. An ethanol precipitation step, as described previously, was performed, and the sample was solubilized in 50 mm ABC, 0.5% SDC. Trypsin was added to a ratio of 1:50 (enzyme:substrate), and the sample was incubated for 16 h at 37 °C. SDC was removed by ethyl acetate extraction under acidic conditions (1 volume of ethyl acetate was added to the sample and acidified with 0.5% TFA). After vigorous vortexing and centrifugation, the organic phase was discarded. An additional step of ethyl acetate extraction without TFA was performed.

**TMT 11 plex labeling**

TMT labeling was performed according to manufactor’s instructions. Samples were resuspended in 100 µL of 200 mM TEAB and the individual TMT reagents were dissolved in 41 µL of dried ACN. Peptides were labeled by mixing the peptide solution with the respective TMT reagent during 1 hour at room temperature. Reaction was quenched by adding 1 µL of 5% hydroxylamine and 15 minutes incubation at room temperature. In each batch/replicate, the labeled peptides were mixed in a single tube, then the volume was reduced in a speed vac and the peptides were cleaned up in a C-18 Sep-Pak cartridge (Waters). After cleaning peptides were dried in a speed vac and finally resuspended in 20 mM ammonium formiate prior to HpH fractionation.

**Peptide fractionation**

Strong cation exchange (SCX)

Peptide mixtures were further fractionated by SCX using Microspin columns (MA SEM HIL-SCX, 10–100μg capacity, The Nest group Inc., South Borough). The peptides were eluted by stepwise salt gradient using 0, 20, 40, 60, 100 and 500 mM KCl in 10 mM potassium phosphate, 20% acetonitrile, pH 2.8.

High pH RP-HPLC

TMT-labelled peptides were separated into 24-25 fractions by basic reversed-phase liquid chromatography on a Phenomenex Aeris C8 column (100 mm × 2.1 mm, 3.6-μm particles) in an Agilent 1100 HPLC system and using a110 minutes gradient. Solvent A: 20 mM ammonium formiate pH 10; solvent B: 20 mM ammonium formiate in 90% ACN. Fractions were dried in a speed vac prior to LC-MS/MS analysis.

**Peptide desalting**

All enzymatic digestions using the buffer exchange, FASP and the MEDFASP methods were quenched by adding formic acid to a final concentration of 1%. The generated proteolytic peptides as well as those eluting from SCX stepwise fractionation were desalted on C18-microcolumns (The Nest Group, MA, USA) following the manufacturer’s instruction. After elution, the peptides were dried on a Speevac, dissolved in 0.1% Formic acid and stored at −20 °C until analysis by mass spectrometry.

Urea in-solution digestions were quenched by adding 20% TFA to a final concentration of ~1%. Peptides were then desalted on the AssayMAP Bravo platform using the peptide cleanup v2.0 protocol. C18 cartridges (Agilent, 5 µL bed volume) were primed with 100 µL 90% acetonitrile (ACN) and equilibrated with 70 µL 0.1% TFA at a flow rate of 10 µL/min. The samples were loaded at 5 µL/min, followed by an internal cartridge wash with 0.1% TFA at a flow rate of 10 µL/min. Peptides were eluted with 30 µL 80% ACN, 0.1% TFA and dried in speed vac and stored at −20 °C until analysis by mass spectrometry or subjected to TMT labeling reaction.

Peptide generated by Arg-C like digestion and digestion on the S-traps were directly analyzed by LC-MS/MS without desalting.

**References**

1-Yakovleva ME, Welinder C, Sugihara Y, et al. Workflow for large-scale analysis of melanoma tissue samples. *EuPA Open Proteomics*. 2015;8:78-84. doi:10.1016/j.euprot.2015.07.011

2- Betancourt LH, Pawłowski K, Eriksson J, et al. Improved survival prognostication of node-positive malignant melanoma patients utilizing shotgun proteomics guided by histopathological characterization and genomic data. *Sci Rep*. 2019;9(1):5154. doi:10.1038/s41598-019-41625-z

3- Betancourt LH, Sanchez A, Pla I, et al. Quantitative Assessment of Urea In-Solution Lys-C/Trypsin Digestions Reveals Superior Performance at Room Temperature over Traditional Proteolysis at 37 °C. *J Proteome Res*. 2018;17(7):2556-2561. doi:10.1021/acs.jproteome.8b00228

4- Kuras M, Betancourt LH, Rezeli M, et al. Assessing Automated Sample Preparation Technologies for High-Throughput Proteomics of Frozen Well Characterized Tissues from Swedish Biobanks. *J Proteome Res*. 2019;18(1):548-556. doi:10.1021/acs.jproteome.8b00792

5- Kuras M, Woldmar N, Kim Y, et al. Proteomic Workflows for High-Quality Quantitative Proteome and Post-Translational Modification Analysis of Clinically Relevant Samples from Formalin-Fixed Paraffin-Embedded Archives. *J Proteome Res*. 2021;20(1):1027-1039. doi:10.1021/acs.jproteome.0c00850

6- Gil J, Ramírez-Torres A, Chiappe D, et al. Lysine acetylation stoichiometry and proteomics analyses reveal pathways regulated by sirtuin 1 in human cells. *J Biol Chem*. 2017. doi:10.1074/jbc.M117.784546
